# Dairy consumption and risk of type 2 diabetes: 3 cohorts of US adults and an updated meta-analysis

**DOI:** 10.1186/s12916-014-0215-1

**Published:** 2014-11-25

**Authors:** Mu Chen, Qi Sun, Edward Giovannucci, Dariush Mozaffarian, JoAnn E Manson, Walter C Willett, Frank B Hu

**Affiliations:** Department of Nutrition, Harvard School of Public Health, Boston, MA USA; Department of Epidemiology, Harvard School of Public Health, Boston, MA USA; Channing Division of Network Medicine, Brigham and Women’s Hospital and Harvard Medical School, Boston, MA USA; Division of Cardiovascular Medicine, and Division of Preventive Medicine, Department of Medicine, Brigham and Women’s Hospital and Harvard Medical School, Boston, MA USA; Division of Preventive Medicine, Department of Medicine, Brigham and Women’s Hospital and Harvard Medical School, Boston, MA USA

**Keywords:** dairy, type 2 diabetes, cohort, meta-analysis

## Abstract

**Background:**

The relation between consumption of different types of dairy and risk of type 2 diabetes (T2D) remains uncertain. Therefore, we aimed to evaluate the association between total dairy and individual types of dairy consumptions and incident T2D in US adults.

**Methods:**

We followed 41,436 men in the Health Professionals Follow-Up Study (1986 to 2010), 67,138 women in the Nurses’ Health Study (1980 to 2010), and 85,884 women in the Nurses’ Health Study II (1991 to 2009). Diet was assessed by validated food-frequency questionnaires, and data were updated every four years. Incident T2D was confirmed by a validated supplementary questionnaire.

**Results:**

During 3,984,203 person-years of follow-up, we documented 15,156 incident T2D cases. After adjustment for age, body mass index (BMI) and other lifestyle and dietary risk factors, total dairy consumption was not associated with T2D risk and the pooled hazard ratio (HR) (95% confidence interval (CI)) of T2D for one serving/day increase in total dairy was 0.99 (0.98, 1.01). Among different types of dairy products, neither low-fat nor high-fat dairy intake was appreciably associated with risk of T2D. However, yogurt intake was consistently and inversely associated with T2D risk across the three cohorts with the pooled HR of 0.83 (0.75, 0.92) for one serving/day increment (*P* for trend <0.001). We conducted a meta-analysis of 14 prospective cohorts with 459,790 participants and 35,863 incident T2D cases; the pooled relative risks (RRs) (95% CIs) were 0.98 (0.96, 1.01) and 0.82 (0.70, 0.96) for one serving total dairy/day and one serving yogurt/day, respectively.

**Conclusions:**

Higher intake of yogurt is associated with a reduced risk of T2D, whereas other dairy foods and consumption of total dairy are not appreciably associated with incidence of T2D.

**Electronic supplementary material:**

The online version of this article (doi:10.1186/s12916-014-0215-1) contains supplementary material, which is available to authorized users.

## Background

Type 2 diabetes (T2D) affects approximately 26 million people in the United States and 366 million people worldwide, and the number will reach an estimated 552 million worldwide by 2030 [1]. Further, management of diabetes and its complications, such as cardiovascular disease, imposes enormous medical and economic burdens [2]. Therefore, primary prevention of T2D has become a public health imperative.

Because of their high content of calcium, magnesium, vitamin D, whey protein and specific fatty acids, dairy products have been suggested to lower risk of T2D [3]. Experimental studies indicated that whey protein has insulinotropic and glucose-lowering properties [4]. Several epidemiologic studies, but not all, have suggested that dairy or calcium consumption was associated with lower risks for hypertension and coronary artery disease [5]. On the other hand, saturated fats in some dairy products might offset the benefits of the potentially protective dairy components [6], whereas other median chain saturated and ruminant trans fatty acids could reduce risk [7]. Total dairy product intake was associated with marginally significantly lower risk of T2D in a recent meta-analysis of prospective cohort studies [8]. However, three large Harvard cohorts, Health Professionals’ Follow-up Study (HPFS), Nurses’ Health Study (NHS) and II, accounting for 38% of participants included in the meta-analysis, have accumulated up to 12 additional years of follow-up since initial publications and the analyses have not been updated. Furthermore, the meta-analysis has insufficient data to evaluate most individual dairy subtypes robustly, especially yogurt, which has been linked to lower obesity and may influence gut microbiota through probiotics [9]. In a previous study [10], yogurt consumption was associated with the least weight gain among all the food types in our cohorts. To address these questions, we aimed to update our previous analyses of total dairy and T2D risk with longer duration of follow-up in the three large cohorts and then assess subtypes of dairy consumption in detail. We also conducted an updated meta-analysis of our results from these three cohorts and previous literature.

## Methods

### Study population

We used data from three prospective cohort studies: HPFS, NHS and NHS II. The HPFS was composed of 51,529 US male dentists, pharmacists, veterinarians, optometrists, osteopathic physicians and podiatrists, 40- to 75-years old, who returned a baseline questionnaire that inquired about detailed medical history, as well as lifestyle and usual diet in 1986. The NHS was initiated in 1976, when 121,700 female registered nurses, 30- to 55-years old, who lived in one of eleven states, completed a baseline questionnaire about their lifestyle and medical history. The NHS II was established in 1989 and consisted of 116,671 younger female registered nurses, 25- to 42-years old, who responded to a baseline questionnaire similar to the NHS questionnaire. Detailed descriptions of the three cohorts have been published elsewhere [11-13]. In all three cohorts, questionnaires were administered at baseline and biennially thereafter, to collect and update information on lifestyle practice and occurrence of chronic diseases. The follow-up rates of the participants in these cohorts were all >90%. In the current analysis, we excluded men and women who had diagnoses of diabetes (including type 1 and type 2 diabetes and gestational diabetes only), cardiovascular disease, or cancer at baseline (1986 for HPFS, 1980 for NHS, and 1991 for NHS II, when we first assessed diet in these cohorts) [14]. In addition, we excluded participants who left >70 of the 131 food items blank on the baseline food frequency questionnaire (FFQ) or who reported unusual total energy intakes (that is, daily energy intake <800 or >4,200 kcal/day for men and <500 or >3,500 kcal/day for women). We also excluded participants without baseline information on dairy consumption or follow-up information on diabetes diagnosis date. After exclusions, data from 41,479 HPFS participants, 67,138 NHS participants and 85,884 NHS II participants were available for analysis. The study protocol was approved by the institutional review boards of Brigham and Women’s Hospital and Harvard School of Public Health. The completion of the self-administered questionnaire was considered to imply informed consent.

### Assessment of dairy consumption

In 1980, a 61-item FFQ was administered to the NHS participants to collect information on their usual intake of foods and beverages in the previous year. In 1984, 1986, 1990, 1994, 1998 and 2002, similar but expanded 131-item FFQs were sent to these participants to update their diet records. With the use of the expanded FFQ used in the NHS, dietary data were collected in 1986, 1990, 1994, 1998 and 2002 from the HPFS participants, and in 1991, 1995, 1999 and 2003 from the NHS II participants.

In all FFQs, we asked the participants how often, on average, they consumed each food of a standard portion size. There were nine possible responses, which ranged from ‘never or less than once per month’ to ‘6 or more times per day’. Nutrient intake was calculated by multiplication of the frequency of consumption of each food by the nutrient composition in the standard portion size of that food and then summing up the nutrient intake from all relevant food items. The food composition database was created primarily from USDA sources [15]. Questionnaire items on dairy products included ‘skim/low fat milk’ , ‘whole milk’ , ‘ice cream’ , ‘yogurt’ , ‘cottage/ricotta cheese’ , ‘cream cheese’ , ‘other cheese’ , ‘cream’. From 1994 in NHS and HPFS and 1995 in NHS II, yogurt consumption was separated into two items, ‘plain yogurt’ (plain or with NutraSweet) and ‘flavored yogurt’ (without NutraSweet). The standard serving size was an 8 oz. glass for skim, low fat milk, or whole milk, 1 Tbs for cream, sour cream, ½ cup for sherbet or frozen yogurt, ice cream, cottage or ricotta cheese, 1 oz. for cream cheese or other cheese. The reproducibility and validity of these FFQs have been shown in detail elsewhere [16-20]. The correlation coefficients between FFQ and multiple dietary records were 0.62 both for low-fat dairy foods and for high-fat dairy foods [20] and ranged from 0.57 for hard cheese to 0.97 for yogurt regarding various dairy products intakes [16].

### Assessment of covariates

In the biennial follow-up questionnaires, we inquired about and updated information on risk factors for chronic diseases, such as body weight, cigarette smoking, physical activity, medication use and family history of diabetes, as well as history of chronic diseases, including hypertension and hypercholesterolemia. Among NHS and II participants, we ascertained menopausal status, postmenopausal hormone use and oral contraceptive use.

### Assessment of diabetes

A supplementary questionnaire about symptoms, diagnostic tests, and hypoglycemic therapy was mailed to participants who reported that they had received a diagnosis of diabetes. In accordance with National Diabetes Data Group criteria, a case of T2D was considered confirmed if at least one of the following was reported on the supplementary questionnaire [21]: 1) one or more classic symptoms (excessive thirst, polyuria, weight loss, hunger) and fasting plasma glucose concentrations ≥7.8 mmol/L or random plasma glucose concentrations ≥11.1 mmol/L; 2) ≥2 elevated plasma glucose concentrations on different occasions (fasting concentrations ≥7.8 mmol/L, random plasma glucose concentrations ≥11.1 mmol/L, and/or concentrations of ≥11.1 mmol/L after ≥2 hours shown by oral-glucose-tolerance testing) in the absence of symptoms; or 3) treatment with hypoglycemic medication (insulin or oral hypoglycemic agent). The diagnostic criteria were changed by the American Diabetes Association in June 1998, and the threshold for the diagnosis of diabetes became a fasting plasma glucose of 7.0 mmol/L, instead of 7.8 mmol/L [22]. Only cases confirmed by the supplemental questionnaires were included. The validity of the supplementary questionnaire for the diagnosis of diabetes has been documented previously. Of the 59 T2D cases in HPFS and 62 cases in NHS who were confirmed by the supplementary questionnaire, 57 (97%) and 61 (98%) were reconfirmed by medical records [23,24]. Deaths were identified by reports from next of kin or postal authorities, or by searching the National Death Index. At least 98% of deaths among the study participants were identified [10].

### Statistical analysis

We calculated each individual’s person-years from the date of return of the baseline questionnaire to the date of diagnosis of T2D, death, or the end of the follow-up (31 January 2010 for HPFS, 30 June 2010 for NHS or 30 June 2009 for NHS II), whichever came first. We used time-dependent Cox proportional hazard regression to estimate the hazard ratio (HR) for dairy consumption in relation to the risk of T2D. Our basic model (Model 1) simultaneously controlled for age, calendar time with updated information at each two-year questionnaire cycle, body mass index (BMI), and total energy intake. Model 2 also adjusted for various potential confounding factors, including race, smoking, physical activity, alcohol consumption, menopausal status and menopausal hormone use (NHS and II participants only), oral contraceptive use (NHS II participants only), family history of diabetes and diagnosed hypertension or hypercholesterolemia at baseline. Model 3 further adjusted for trans-fat, glycemic load, and intakes of red and processed meat, nuts, sugar-sweetened beverages (SSBs) and coffee. For individual dairy foods, we additionally adjusted for other types of dairy in model 3.

We used the cumulative average of dietary intakes from baseline to the censoring events in order to best represent long-term diet and minimize within-person variation [14]. In our primary analysis we stopped updating dietary intake when participants developed coronary heart disease, stroke or cancer because changes in diet after development of these conditions may confound the relationship between diet and diabetes [14,25]. We conducted a further analysis by stopping updating dietary information after self-reported diagnosis of hypertension and hypercholesterolemia during the follow-up because these diagnoses appeared to alter consumption of dairy products (see Results).

Proportional hazards assumption was tested with a time dependent variable with the inclusion of an interaction term between the dairy intake and months to events (*P* >0.05 for all tests). To test for linear trend, the median value was assigned to each quintile and this value was modeled as a continuous variable. All the analyses were conducted separately in each cohort, and we also conducted meta-analyses to summarize the estimates of association across the three studies. No significant heterogeneities were shown when the results were pooled across the three cohorts; therefore, fixed-effect models were used. All statistical tests were two-sided and performed using SAS version 9.2 for UNIX (SAS Institute Inc, Cary, NC, USA).

### Updated meta-analysis on dairy products and risk of incident T2D

We further conducted an updated meta-analysis that incorporated our new results from the three cohorts into the findings of previous studies. This meta-analysis was conducted following a review protocol [26]. For study selection, we included prospective studies with cohort, case cohort or nested case-control design investigating the association between intake of dairy products and risk of T2D. The two recent meta-analyses involved a search of the literature up to March 2013 [27] to June 2013 [8]. Thus, we conducted additional literature searches on MEDLINE [28] and EMBASE [29] from June 2013 to October 2013 [see Additional file 1]. In studies that reported the intakes by grams, we used 177 g as a serving size for total dairy products, and 244 g as a serving size for milk and yogurt intake to recalculate the intakes to a common scale (servings/day).

## Results

We documented a total of 15,156 cases of incident diabetes mellitus (DM), including 3,364 cases during a maximum of 24 years of follow-up in the HPFS, 7,841 cases during a maximum of 30 years in the NHS, and 3,951 cases during a maximum of 16 years in the NHS II. For both men and women, total dairy intake was inversely associated with smoking, hypertension and hypercholesterolemia, but positively associated with physical activity, and fruit and vegetable intakes (Table 1). Different types of dairy products were moderately correlated (Spearman correlation coefficients from -0.13 to 0.27 in the three cohorts).

**Table 1:** **Baseline age-adjusted characteristics of participants in the three cohorts according to quintile of total dairy consumption** ^**a**^

	**HPFS (1986)**			**NHS I (1980)**			**NHS II (1991)**		
**Characteristics**	**Q1 (number = 8,638)**	**Q3 (number = 8,823)**	**Q5 (number = 8,323)**	**Q1 (number = 13,456)**	**Q3 (number = 13,397)**	**Q5 (number = 13,433)**	**Q1 (number = 17,225)**	**Q3 (number = 17,147)**	**Q5 (number = 17,213)**
Total dairy intake (servings/day)	0.6 (0.4 to 0.7)^b^	1.6 (1.4 to 1.7)	3.8 (3.4 to 4.6)	0.9 (0.7 to 1.1)	1.9 (1.8 to 2.0)	3.4 (3.1 to 3.8)	0.8 (0.5 to 1.1)	2.0 (1.7 to 2.3)	3.9 (3.4 to 4.8)
Age (years)	52.6(9.1)^c^	52.9(9.5)	53.4(9.7)	46.4 (7.1)	46.1(7.1)	46.3(7.3)	36.8(4.6)	36.0(4.7)	35.2(4.6)
Physical activity (MET-hours/week)	19.9(29.1)	21.6(28.0)	22.1(29.9)	12.3(17.5)	14.0(19.4)	15.4 (21.1)	19.0(27.2)	21.3(26.9)	22.7(28.6)
BMI (kg/m^2^)	24.8(4.9)	25.0(4.8)	25.0(4.9)	24.0(4.3)	24.3(4.3)	24.3(4.4)	24.4(5.3)	24.6(5.3)	24.5(5.1)
Race, white (%)	91.7	96.0	97.0	95.8	98.5	98.9	93.0	97.6	97.9
Current smoker (%)	10.3	8.6	10.5	34.6	25.9	24.0	15.6	10.7	10.8
Hypertension (%)	21.0	19.6	17.4	16.4	14.7	13.7	6.5	6.0	5.2
High cholesterol (%)	12.6	10.0	8.2	6.1	4.7	4.8	16.0	13.6	12.6
Family history of diabetes (%)	23.1	24.7	23.9	28.2	28.3	28.6	34.9	34.1	32.3
Postmenopausal (%)	NA	NA	NA	32.1	31.2	31.3	3.55	2.99	2.72
Current menopausal hormone use (%)^d^	NA	NA	NA	21.6	22.1	21.6	83.1	80.9	83.8
Current oral conceptive use (%)	NA	NA	NA	NA	NA	NA	11.49	12.06	9.14
Total energy (Kcal/day)	1,657(523)	1,957(546)	2,425(626)	1,433(364)	1,699(363)	2,013(403)	1,443(475)	1,791(469)	2,180(514)
Alcohol (g/day)	12.3(16.6)	11.5(15.0)	10.4(15.0)	7.1(11.0)	6.1 (8.8)	5.2(8.0)	3.2(6.6)	3.4(6.0)	3.1(6.0)
Cereal fiber (g/day)	5.6(4.0)	6.0(3.8)	5.6(3.5)	4.5 (2.1)	5.0 (2.1)	4.8 (1.9)	5.4 (3.1)	5.8(3.0)	5.4(2.8)
Glycemic load	127(30)	125(24)	120(230)	103 (20)	103 (16)	101 (15)	127 (26)	121 (20)	115 (18)
Polyunsaturated to saturated fat ratio	0.68(0.26)	0.56(0.17)	0.47(0.15)	0.59(0.18)	0.56 (0.14)	0.51 (0.13)	0.60(0.19)	0.52(0.13)	0.45(0.12)
Trans fat (% of total energy)	2.8 (1.3)	2.9 (1.1)	2.8 (1.0)	1.8 (0.6)	1.7 (0.5)	1.7 (0.4)	1.8(0.7)	1.6(0.6)	1.5(0.5)
Fruit and vegetables (servings/day)	4.9(2.8)	5.4(2.6)	5.8(2.9)	4.3 (1.9)	5.2 (1.9)	5.9 (2.1)	4.1(2.7)	5.2(2.7)	6.0(3.1)
Red and processed meat intake (servings/day)	0.99(0.79)	1.16(0.79)	1.33(0.90)	1.02(0.60)	1.01 (0.51)	1.07 (0.56)	1.03(0.67)	1.17(0.68)	1.24(0.72)
Nuts intake (servings/day)	0.38(0.54)	0.46(0.59)	0.58(0.73)	0.13 (0.18)	0.16(0.19)	0.19 (0.21)	0.07(0.20)	0.09(0.19)	0.10(0.22)
SSB intake (servings/day)	0.34(0.64)	0.36(0.58)	0.40(0.62)	0.33 (0.57)	0.27 (0.42)	0.29 (0.42)	0.54(0.99)	0.45(0.80)	0.45(0.77)
Coffee intake (servings/day)	1.87(1.78)	1.92(1.75)	2.05(1.90)	2.08(1.57)	2.14 (1.42)	2.21 (1.50)	1.43 (1.71)	1.57(1.64)	1.70(1.74)

^a^Data were age standardized except for age and dairy intake. ^b^Median; interquartile range in parentheses (all such values). ^c^Mean SD (all such values). ^d^Current menopausal hormone users among postmenopausal women. HPFS, Health Professionals Follow-Up Study; METs, metabolic equivalent; NA, not available; NHS, Nurses’ Health Study; Q, quintile.

**Figure 1:**
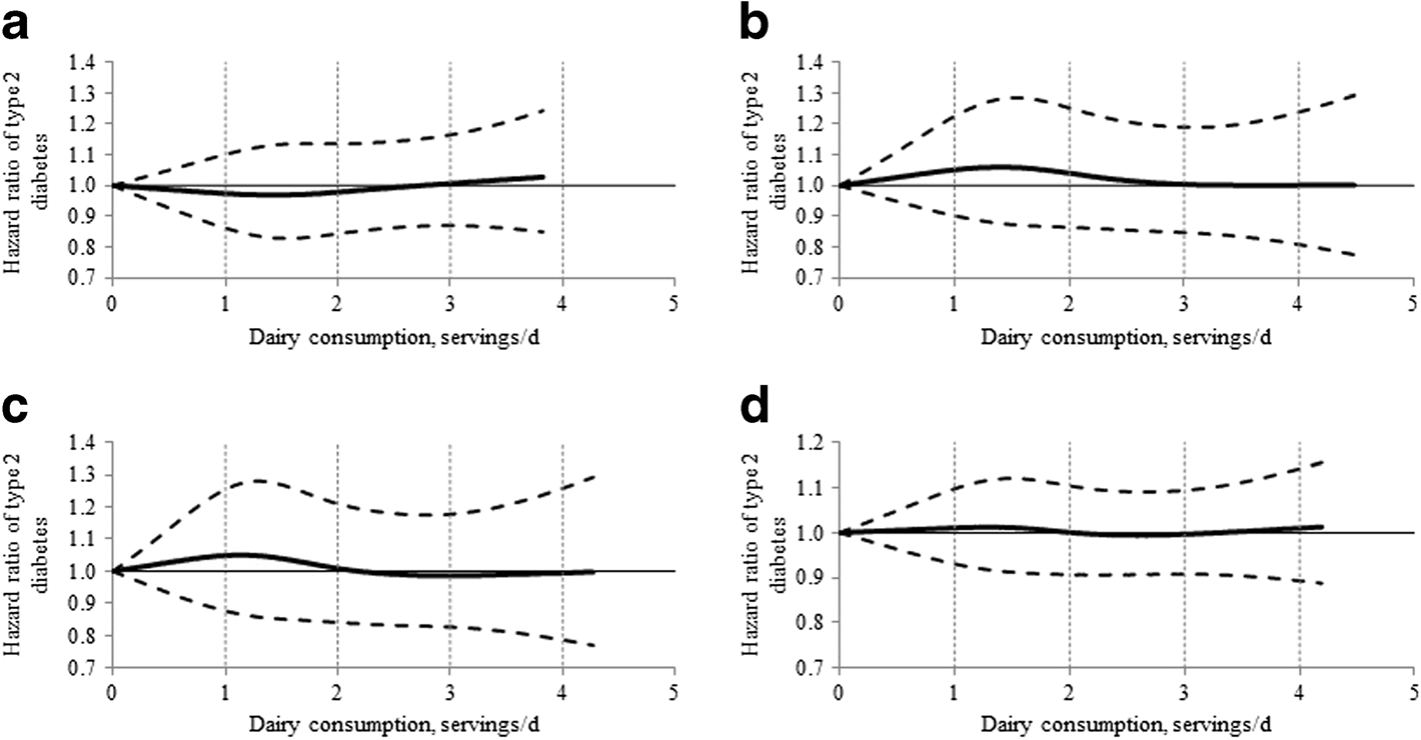
**Dose-response relationship between total dairy intake and risk of type 2 diabetes in HPFS, NHS I and NHS II using multivariate model. a)** NHS. **b)** NHS II. **c)** HPFS. **d)** Pooled. HPFS, Health Professionals Follow-up Study; NHS, Nurses Health Study.

Total dairy consumption was not associated with risk of T2D in age- and multivariate-adjusted models across the three cohorts (all *P* for trend >0.05), as shown in Table 2. In the pooled analysis of estimates from the three studies that used fixed-effect models, in the age-, BMI- and energy-adjusted model, one serving/day increment of dairy consumption was significantly associated with a 4% lower risk (95% confidence interval (CI): 2%, 6%); however, further adjustment for lifestyle and other dietary factors attenuated the association to null with the HR of a one serving/day increase of 0.99 (95% CI: 0.98, 1.01). The cohort-specific and combined spline analyses (Figure 1) based on multivariate models also indicated a null association between total dairy consumption and T2D risk. No interactions of total dairy consumption with age, BMI, vitamin D level, physical activity level and diabetes family history were observed [see Additional file 1: Table S1].

**Table 2:** **HRs (95% CI) of type 2 diabetes risk according to quintile of total dairy intake in HPFS, NHS I and NHS II**

	**Frequency of consumption**					***P*** **-trend** ^**b**^	**HR (95% CI) for one serving/day**
	**Q1** ^**a**^	**Q2** ^**a**^	**Q3** ^**a**^	**Q4** ^**a**^	**Q5** ^**a**^		
HPFS							
Daily servings	(0.64, <0.86)^c^	(1.18, 0.86 to 1.34)	(1.64, 1.35 to 1.90)	(2.32, 1.91, 2.99)	(3.59, ≥3.00)		
Cases/person-years	660/161,017	698/157,956	654/159,981	669/160.184	683/159,307		
Age-, BMI-and energy-adjusted^d^	1.0	1.06 (0.95, 1.18)	0.98 (0.87, 1.09)	0.97 (0.87, 1.09)	0.97 (0.87, 1.09)	0.34	0.98 (0.95, 1.01)
Adjusted for non-dietary factors^d^	1.0	1.10 (0.98, 1.22)	1.02 (0.92, 1.14)	1.00 (0.89, 1.12)	1.01 (0.90, 1.14)	0.60	0.99 (0.96, 1.02)
Adjusted for dietary factors^d^	1.0	1.08 (0.97, 1.21)	1.01 (0.91, 1.13)	0.99 (0.88, 1.11)	0.99 (0.88, 1.11)	0.38	0.98 (0.95, 1.01)
NHS							
Daily servings	(0.91, <1.20)	(1.45, 1.20 to 1.66)	(1.91, 1.67 to 2.15)	(2.45, 2.16, 2.83)	(3.37, ≥2.84)		
Cases/person-years	1,596/350,963	1,575/350,493	1,531/351,353	1,584/350,639	1,555/351,058		
Age-, BMI-and energy-adjusted	1.00	0.93 (0.87, 1.00)	0.88 (0.82, 0.95)	0.90 (0.84, 0.97)	0.90 (0.83, 0.97)	0.01	0.97 (0.94, 0.99)
Adjusted for non-dietary factors	1.00	0.97 (0.90, 1.04)	0.93 (0.86, 1.00)	0.94 (0.88, 1.02)	0.94 (0.87, 1.02)	0.13	0.98 (0.95, 1.00)
Adjusted for dietary factors	1.00	1.00 (0.93, 1.07)	0.98 (0.91, 1.06)	1.03 (0.95, 1.11)	1.05 (0.97, 1.14)	0.15	1.02 (0.99, 1.05)
NHS II							
Daily servings	(0.79, <1.06)	(1.40, 1.06 to 1.61)	(1.96, 1.62 to 2.24)	(2.71, 2.25, 3.02)	(3.85, ≥3.03)		
Cases/person-years	856/283,968	866/285,509	802/286,713	731/286,865	696/288,188		
Age-, BMI-and energy-adjusted	1.00	1.00 (0.91, 1.10)	0.92 (0.83, 1.01)	0.85 (0.77, 0.95)	0.83 (0.75, 0.92)	<0.001	0.93 (0.90, 0.96)
Adjusted for non-dietary factors	1.00	1.05 (0.96, 1.16)	1.01 (0.91, 1.11)	0.95 (0.86, 1.06)	0.94 (0.84, 1.04)	0.05	0.96 (0.94, 0.99)
Adjusted for dietary factors	1.00	1.08 (0.98, 1.18)	1.04 (0.94, 1.15)	0.99 (0.89, 1.10)	1.00 (0.89, 1.11)	0.46	0.98 (0.95, 1.01)
Pooled analysis							
Age-, BMI-and energy-adjusted	1.00	0.98 (0.93, 1.03)	0.91 (0.86, 0.96)	0.90 (0.86, 0.95)	0.90 (0.85, 0.95)	<0.001^e^	0.96 (0.94, 0.98)^e^
Adjusted for non-dietary factors	1.00	1.02 (0.97, 1.07)	0.97 (0.92, 1.02)	0.96 (0.91, 1.01)	0.95 (0.90, 1.01)	0.02	0.98 (0.96, 0.99)
Adjusted for dietary factors	1.00	1.04 (0.98, 1.09)	1.00 (0.95, 1.06)	1.01 (0.96, 1.07)	1.02 (0.96, 1.08)	0.99	0.99 (0.98, 1.01)

^a^Q is quintile; ^b^ *P*-trend was calculated by assigning median values to each quintile and was treated as continuous variable; ^c^quintile median and cut-points in parentheses (all such values); ^d^model was adjusted for age (continuous), BMI (eight categories), total energy intake (quintiles). Model 2 was additionally adjusted for race, smoking, physical activity, alcohol consumption, menopausal status and menopausal hormone use (NHS I and II participants only), oral contraceptive use (NHS II participants only), diabetes family history, hypertension, hypercholesterolemia. Model 3 was additionally adjusted for trans-fat intake, glycemic load, red and processed meat intake, nuts intake, SSB intake, and coffee intake. ^e^ *P* for heterogeneity <0.05. BMI, body mass index; CI, confidence interval; HPFS, Health Professionals Follow-Up Study; HRs, hazard ratios; NHS, Nurses’ Health Study; Q, quintile.

When we examined the association with dairy products categorized by their fat contents, there were no significant associations between either low-fat or high-fat dairy intake and risk of T2D (Table 3). The associations between individual types of dairy products and risk of T2D were further assessed, as shown in Table 4. All subtypes of dairy products were mutually adjusted for each other in the multivariate models. In the pooled analysis of estimates from the three studies that used fixed-effects models, each one serving/day increase of skim milk, cheese and whole milk was associated with a 2% (95% CI: -1%, 4%), 7% (95% CI: 3%, 11%) and 10% (95% CI: 4%, 16%) higher risk of T2D, respectively (All *P* for trend <0.05). Conversely, greater yogurt and ice cream intakes were significantly associated with lower risk of T2D with an HR of 0.83 (95% CI: 0.75, 0.92) and 0.78 (95% CI: 0.71, 0.86), respectively. No significant interaction of yogurt consumption with baseline BMI was observed [see Additional file 1: Table S3]. In addition, higher consumption of either plain (HR for each serving: 0.96, 95%CI: 0.88, 1.06) or flavored yogurt (HR: 0.88, 95%CI: 0.77, 1.01) was associated with a nonsignificantly lower risk of T2D.

**Table 3:** **Relative risk (RR) of type 2 diabetes among men according to low-fat versus high-fat dairy food intake**

	**Dairy intake (daily servings)**						**HR (95% CI) for one serving/day**
**Variable**	**Q1** ^**a**^	**Q2** ^**a**^	**Q3** ^**a**^	**Q4** ^**a**^	**Q5** ^**a**^	***P*** **-trend** ^**b**^	
	**Low-fat dairy intake**						
**HPFS**							
Daily servings	(0.07, <0.14)^c^	(0.43, 0.14 to 0.49)	(0.86, 0.50 to 0.99)	(1.28, 1.00 to 1.49)	(2.57, ≥1.50)		
Cases/person-years	709/154,402	681/161,216	666/163,722	663/159,761	645/159,343		
Age-, BMI-and energy-adjusted^d^	1.00	0.93 (0.84, 1.04)	0.89 (0.80, 0.99)	0.92 (0.82, 1.02)	0.88 (0.79, 0.99)	0.06	0.97 (0.93, 1.00)
Unadjusted for dietary factors^d^	1.00	0.95 (0.85, 1.06)	0.92 (0.83, 1.02)	0.94 (0.84, 1.05)	0.90 (0.80, 1.00)	0.09	0.97 (0.93, 1.00)
Adjusted for dietary factors^d^	1.00	0.97 (0.87, 1.07)	0.95 (0.85, 1.06)	0.98 (0.88, 1.09)	0.95 (0.84, 1.06)	0.45	0.98 (0.95, 1.02)
**NHS**							
Daily servings	(0.23, <0.42)	(0.64, 0.43 to 0.82)	(1.03, 0.83 to 1.21)	(1.50, 1.22 to 1.85)	(2.42, ≥1.86)		
Cases/person-years	1,652/351,416	1,504/350,553	1,620/350,912	1,479/350,718	1,586/350,907		
Age-, BMI-and energy-adjusted	1.00	0.89 (0.83, 0.96)	0.93 (0.87, 1.00)	0.84 (0.78, 0.90)	0.90 (0.84, 0.96)	0.004	0.96 (0.94, 0.99)
Unadjusted for dietary factors	1.00	0.93 (0.87, 1.00)	0.97 (0.91, 1.04)	0.89 (0.83, 0.96)	0.93 (0.87, 1.00)	0.06	0.98 (0.95, 1.00)
Adjusted for dietary factors	1.00	0.97 (0.91, 1.04)	1.03 (0.96, 1.10)	0.98 (0.91, 1.06)	1.05 (0.97, 1.14)	0.17	1.02 (0.99, 1.06)
**NHS II**							
Daily servings	(0.21, <0.34)	(0.67, 0.35 to 0.87)	(1.10, 0.88 to 1.21)	(1.68, 1.22 to 2.01)	(2.75, >2.02)		
Cases/person-years	848/283,696	871/286,724	789/287,700	749/286,955	694/286,167		
Age-, BMI-and energy-adjusted	1.00	1.00 (0.91, 1.10)	0.90 (0.82, 0.99)	0.88 (0.79, 0.97)	0.82 (0.74, 0.91)	<0.001	0.92 (0.89, 0.96)
Unadjusted for dietary factors	1.00	1.05 (0.95, 1.15)	0.99 (0.89, 1.09)	0.96 (0.87, 1.06)	0.90 (0.81, 1.00)	0.01	0.95 (0.92, 0.99)
Adjusted for dietary factors	1.00	1.07 (0.97, 1.17)	1.03 (0.93, 1.13)	1.01 (0.91, 1.12)	0.95 (0.85, 1.06)	0.13	0.97 (0.93, 1.01)
**Pooled**							
Multivariate RR (95% CI)	1.00	1.00 (0.95, 1.05)	1.01 (0.96, 1.06)	0.99 (0.94, 1.04)	1.00 (0.94, 1.05)	0.72	1.00 (0.98, 1.02)^e^
	**High-fat dairy intake**						
**HPFS**							
Daily servings	(0.16, <0.28)	(0.42, 0.28 to 0.49)	(0.64, 0.50 to 0.72)	(0.97, 0.73 to 1.14)	(1.78, >1.15)		
Cases/person-years	621/159,021	615/161,726	695/159,337	694/159,123	739/159,238		
Age-, BMI-and energy-adjusted	1.00	0.92 (0.82, 1.03)	1.02 (0.92, 1.14)	0.97 (0.86, 1.08)	1.04 (0.93, 1.16)	0.24	1.01 (0.96, 1.05)
Unadjusted for dietary factors	1.00	0.95 (0.85, 1.06)	1.08 (0.96, 1.20)	1.03 (0.92, 1.15)	1.09 (0.98, 1.23)	0.05	1.02 (0.97, 1.06)
Adjusted for dietary factors	1.00	0.90 (0.80, 1.01)	0.99 (0.88, 1.11)	0.94 (0.84, 1.05)	0.97 (0.86, 1.09)	0.87	0.98 (0.94, 1.03)
**NHS**							
Daily servings	(0.21, <0.31)	(0.42, 0.32 to 0.50)	(0.62, 0.51 to 0.73)	(0.89, 0.74 to 1.10)	(1.53, >1.11)		
Cases/person-years	1,620/351,081	1,557/350,717	1,530/350,871	1,551/350,817	1,583/351,020		
Age-, BMI-and energy-adjusted	1.00	0.92 (0.86, 0.98)	0.89 (0.83, 0.96)	0.89 (0.82, 0.95)	0.96 (0.89, 1.04)	0.83	1.01 (0.97, 1.05)
Unadjusted for dietary factors	1.00	0.95 (0.88, 1.02)	0.93 (0.86, 1.00)	0.92 (0.86, 0.99)	0.99 (0.92, 1.07)	0.80	1.01 (0.97, 1.05)
Adjusted for dietary factors	1.00	0.95 (0.89, 1.02)	0.93 (0.86, 1.00)	0.92 (0.85, 0.99)	1.01 (0.93, 1.09)	0.51	1.03 (0.99, 1.07)
**NHS II**							
Daily servings	(0.20, <0.28)	(0.43, 0.29 to 0.52)	(0.64, 0.53 to 0.77)	(0.98, 0.78 to 1.17)	(1.71, >1.18)		
Cases/person-years	792/289,456	775/274,246	843/293,070	811/286,254	730/288,217		
Age-, BMI-and energy-adjusted	1.00	0.95 (0.86, 1.05)	1.01 (0.91, 1.11)	1.01 (0.91, 1.12)	0.94 (0.85, 1.05)	0.40	0.96 (0.92, 1.01)
Unadjusted for dietary factors	1.00	0.98 (0.89, 1.09)	1.07 (0.97, 1.19)	1.09 (0.98, 1.20)	1.02 (0.92, 1.14)	0.49	0.99 (0.94, 1.03)
Adjusted for dietary factors	1.00	0.97 (0.88, 1.07)	1.05 (0.95, 1.16)	1.07 (0.96, 1.18)	1.03 (0.92, 1.15)	0.35	1.00 (0.95, 1.05)
**Pooled**							
Multivariate Model	1.00	0.94 (0.90, 0.99)	0.97 (0.92, 1.02)	0.96 (0.91, 1.01)	1.00 (0.95, 1.06)	0.30	1.01 (0.98, 1.03)

^a^Q is quintile; ^b^ *P*-trend was calculated by assigning median values to each quintile and was treated as continuous variable; ^c^quintile median and cut-points in parentheses (all such values); ^d^Model 1 was adjusted for age (continuous), BMI (eight categories), total energy intake (quintiles). Model 2 was additionally adjusted for race, smoking, physical activity, alcohol consumption, menopausal status and menopausal hormone use (NHS I and II participants only), oral contraceptive use (NHS II participants only), diabetes family history, hypertension, hypercholesterolemia. Model 3 was additionally adjusted for trans-fat intake, glycemic load, red and processed meat intake, nuts intake, SSB intake, and coffee intake. ^e^ *P* for heterogeneity <0.05. BMI, body mass index; CI, confidence interval; HPFS, Health Professionals Follow-up Study; NHS, Nurses Health Study.

**Table 4:** **Multivariate relative risk (RR) of type 2 diabetes among men and women according to intakes of dairy foods**

	**Dairy intake (servings)**				***P*** **for trend** ^**a**^	**HR (95% CI) for one serving/day**
**Variable**	**Category 1**	**Category 2**	**Category 3**	**Category 4**		
**Skim/low-fat milk**	<1/week	1 to 4/week	5/week to 1.4/day	≥1.5/day		
HPFS	0/week^c^	2.3/week	0.9/day	2.5/day		
Cases/person-years	800/187,455	878/211,190	1,095/263,155	591/136,644		
Multivariate model^*b*^	1.00	1.01 (0.91, 1.11)	1.05 (0.96, 1.16)	1.05 (0.94, 1.18)	0.35	0.99 (0.96, 1.03)
NHS	0.2/week	2.6/week	1.0/day	2.4/day		
Cases/person-years	1,434/324,753	2,022/478,343	2,932/652,108	1,453/299,303		
Multivariate model	1.00	1.00 (0.93, 1.08)	1.05 (0.98, 1.12)	1.13 (1.04, 1.23)	<0.001	1.06 (1.02, 1.09)
NHS II	0.2/week	3.0/week	1.0/day	2.5/day		
Cases/person-years	629/222,580	1,181/414,562	1,490/521.220	651/272,879		
Multivariate model	1.00	1.05 (0.95, 1.16)	1.15 (1.04, 1.27)	1.02 (0.91, 1.15)	0.92	0.97 (0.93, 1.01)
Pooled						
Multivariate model	1.00	1.02 (0.97, 1.07)	1.07 (1.02, 1.12)	1.08 (1.02, 1.15)	0.006^*d*^	1.02 (0.99, 1.04)^d^
**Cheese intake**	<1/week	1 to 4/week	5/week to 1.4/day	≥1.5/day		
HPFS	0.6/week	2.6/week	0.8/day	1.8/day		
Cases/person-years	407/110,338	1,658/415,482	1,167/250,081	132/22,544		
Multivariate Model	1.00	1.07 (0.96, 1.20)	1.16 (1.02, 1.31)	1.31 (1.07, 1.62)	0.002	1.07 (0.99, 1.15)
NHS	0.7/week	2.6/week	0.8/day	2.3/day		
Cases/person-years	850/166,664	4,223/990,161	2,623/568,288	145/29,393		
Multivariate Model	1.00	0.95 (0.90, 0.99)	1.00 (0.97, 1.04)	1.06 (0.90, 1.26)	0.09	1.09 (1.02, 1.17)
NHS II	0.7/week	2.6/week	0.8/day	1.7/day		
Cases/person-years	364/118,132	1,964/751,355	1,499/520,280	124/41,475		
Multivariate Model	1.00	0.90 (0.80, 1.02)	0.97 (0.85, 1.10)	0.89 (0.72, 1.10)	0.80	1.04 (0.96, 1.12)
Pooled						
Multivariate Model	1.00	0.96 (0.92, 1.00)^*d*^	1.01 (0.98, 1.05)^*d*^	1.08 (0.96, 1.20)^*d*^	0.004	1.07 (1.03, 1.11)
**Yogurt**	<1/month	1 to 3/month	1/week	≥2/week		
HPFS	0/month	2.1/month	1.0/week	3.0/week		
Cases/person-years	1,894/413,496	669/160,207	485/134,656	316/90,085		
Multivariate model	1.00	0.97 (0.88, 1.06)	0.89 (0.80, 0.99)	0.95 (0.84, 1.08)	0.30	0.85 (0.68, 1.06)
NHS	0/month	1.8/month	1.2/week	2.9/week		
Cases/person-years	3,118/608,342	1,762/360,994	1,930/476,985	1,031/308,184		
Multivariate model	1.00	1.02 (0.96, 1.08)	0.91 (0.85, 0.97)	0.84 (0.78, 0.91)	<0.001	0.75 (0.65, 0.86)
NHS II	0/month	2.1/month	1.0/week	2.7/week		
Cases/person-years	1,153/354,086	867/299,194	1,174/438,017	757/339,945		
Multivariate model	1.00	1.00 (0.91, 1.09)	1.00 (0.91, 1.09)	0.90 (0.81, 1.00)	0.02	0.94 (0.80, 1.10)
Pooled						
Multivariate model	1.00	1.00 (0.96, 1.05)	0.93 (0.89, 0.97)	0.88 (0.83, 0.93)	<0.001	0.83 (0.75, 0.92)
**Whole milk**	<1/month	1 to 3/month	1/week	≥2/week		
HPFS	0/month	2.1/month	1.5/week	5.2/week		
Cases/person-years	2,492/606,198	323/70,991	215/51,858	334/69,398		
Multivariate model	1.00	1.05 (0.93, 1.18)	0.96 (0.83, 1.11)	1.11 (0.98, 1.25)	0.13	1.07 (0.98, 1.17)
NHS	0/month	1.5/month	1.05/week	3.5/week		
Cases/person-years	4,736/107,7815	959/207,604	1,091/245,503	1,055/223,583		
Multivariate model	1.00	1.01 (0.94, 1.09)	0.99 (0.93, 1.06)	1.08 (1.00, 1.15)	0.05	1.14 (1.06, 1.23)
NHS II	0/month	2.1/month	1.5/week	4.7/week		
Cases/person-years	3,373/122,3599	242/84,815	190/66,854	146/55,973		
Multivariate model	1.00	0.96 (0.84, 1.09)	1.04 (0.90, 1.21)	1.03 (0.87, 1.22)	0.65	0.98 (0.83, 1.14)
Pooled						
Multivariate model	1.00	1.01 (0.96, 1.07)	1.00 (0.94, 1.05)	1.08 (1.02, 1.14)	0.02	1.10 (1.04, 1.16)
**Ice Cream**	<1/month	1 to 3/month	1/week	≥2/week		
HPFS	0/month	2.1/month	1.0/week	3.0/week		
Cases/person-years	778/165,934	1,262/287,954	851/218,100	473/126,457		
Multivariate RR (95% CI)	1.00	0.83 (0.75, 0.91)	0.68 (0.62, 0.76)	0.63 (0.56, 0.71)	<0.001	0.84 (0.71, 0.99)
NHS	0.6/month	1.8/month	1.1/week	2.8/week		
Cases/person-years	1,282/286,271	2,822/626,515	2,518/570,309	1,219/271,411		
Multivariate RR (95% CI)	1.00	0.90 (0.84, 0.96)	0.83 (0.78, 0.89)	0.79 (0.72, 0.86)	<0.001	0.71 (0.62, 0.82)
NHS II	0/month	2.1/month	1.1/week	3.0/week		
Cases/person-years	1,185/436,526	1,679/598,547	830/306,473	257/89,696		
Multivariate RR (95% CI)	1.00	0.88 (0.82, 0.95)	0.77 (0.70, 0.84)	0.79 (0.69, 0.91)	<0.001	0.90 (0.73, 1.12)
Pooled						
Multivariate RR (95% CI)	1.00	0.87 (0.84, 0.91)	0.78 (0.74, 0.82)^*d*^	0.74 (0.70, 0.79)^*d*^	<0.001^*d*^	0.78 (0.71, 0.86)
**Cream**	<1/month	1 to 3/month	1/week	≥2/week		
HPFS	0/month	1.5/month	1.0/week	4.5/week		
Cases/person-years	2,145/501,934	509/127,177	261/66,686	449/102,648		
Multivariate model	1.00	0.95 (0.86, 1.05)	0.94 (0.82, 1.07)	0.98 (0.88, 1.09)	0.84	0.94 (0.87, 1.02)
NHS	0/month	1.8/month	1.1/week	4.8/week		
Cases/person-years	4,271/822,910	1,257/269,337	820/178,495	956/212,976		
Multivariate model	1.00	0.96 (0.90, 1.03)	0.92 (0.85, 0.99)	0.99 (0.92, 1.07)	0.92	1.01 (0.95, 1.07)
NHS II	0/month	1.5/month	0.7/week	3.5/week		
Cases/person-years	1,703/590,798	874/305,399	618/215,329	756/319,716		
Multivariate model	1.00	1.00 (0.92, 1.09)	1.02 (0.93, 1.13)	1.04 (0.94, 1.14)	0.46	1.02 (0.95, 1.09)
Pooled						
Multivariate model	1.00	0.97 (0.93, 1.02)	0.95 (0.90, 1.01)	1.00 (0.95, 1.05)	0.88	0.99 (0.95, 1.03)
**Sherbet**	<1/month	1-3/month	1/week	≥2/week		
HPFS	0/month	1.8/month	1.0/week	2.4/week		
Cases/person-years	1,386/325,655	1,050/251,524	558/136,755	370/84,511		
Multivariate model	1.00	1.01 (0.93, 1.10)	1.05 (0.94, 1.17)	1.09 (0.97, 1.23)	0.13	0.98 (0.80, 1.21)
NHS	0.1/month	1.8/month	1.1/week	2.8/week		
Cases/person-years	3,052/609,546	2,081/402,796	1,576/335,068	595/136,309		
Multivariate model	1.00	1.06 (1.00, 1.13)	1.02 (0.95, 1.09)	1.00 (0.91, 1.10)	0.79	0.96 (0.82, 1.12)
NHS II	0/month	2.1/month	1.3/week	3.0/week		
Cases/person-years	1,292/501,292	1,413/499,003	946/321,704	300/109,243		
Multivariate model	1.00	1.03 (0.95, 1.11)	1.00 (0.92, 1.10)	0.93 (0.81, 1.06)	0.24	0.94 (0.78, 1.14)
Pooled						
Multivariate model	1.00	1.04 (1.00, 1.09)	1.02 (0.97, 1.07)	1.01 (0.94, 1.07)	0.88	0.96 (0.87, 1.07)

^a^ *P*-trend was calculated by assigning median values to each quintile and was treated as continuous variable; ^b^multivariate model was adjusted for age (continuous), BMI (eight categories), total energy intake (quintiles), race, smoking, physical activity, alcohol consumption, menopausal status and menopausal hormone use (NHS I and II participants only), oral contraceptive use (NHS II participants only), diabetes family history, hypertension, hypercholesterolemia, trans-fat intake, glycemic load, red and processed meat intake, nuts intake, SSB intake, and coffee intake, and other dairy types for individual dairy types; ^c^category median intake (all such values); ^d^ *P* for heterogeneity <0.05. CI, confidence interval; HPFS, Health Professionals Follow-up Study; HR, hazard ratio; NHS, Nurses Health Study.

We conducted a further analysis by additionally stopping updating dietary information after self-reported diagnosis of hypertension or hypercholesterolemia during the follow-up as the consumption of ice-cream was decreased but consumption of skim milk was increased after diagnosis of hypertension or hypercholesterolemia in our three cohorts [see Additional file 1: Table S4]. As shown in Table 5, the significant associations between skim milk, cheese, whole milk and risk of T2D became null, with the corresponding HRs of 1.01 (95%CI: 0.99, 1.03), 1.03 (95%CI: 0.99, 1.07) and 1.03 (95%CI: 0.99, 1.07), respectively (all *P*-trend >0.05). The inverse association between ice cream and T2D risk attenuated with an HR of 0.89 (95%CI: 0.83, 0.96), although still significant. On the contrary, the inverse association between yogurt intake and risk of T2D remained significant with an HR of 0.86 (95%CI: 0.78, 0.94) for one serving per day increment.

**Table 5:** **Multivariate relative risk (RR) of type 2 diabetes among men and women according to specific dairy foods using different methods of updating diets**

	**Dairy Intake (servings)**				***P*** **for trend** ^**a**^	**HR (95% CI) for one serving/day**
**Variable**	**Category 1**	**Category 2**	**Category 3**	**Category 4**		
**Skim/low-fat milk**	<1/week	1 to 4/week	5/week to 1.4/day	≥1.5/day		
Direct update^b^	1.00	1.11 (1.05, 1.17)	1.16 (1.10, 1.22)	1.19 (1.12, 1.26)	<0.001	1.02 (1.00, 1.05)
Stop updating CVD and cancer	1.00	1.02 (0.97, 1.07)	1.07 (1.02, 1.12)	1.08 (1.02, 1.15)	0.006	1.02 (0.99, 1.04)
Stop updating + HTHC^c^	1.00	0.94 (0.90, 0.99)	0.97 (0.93, 1.01)	1.02 (0.97, 1.07)	0.25	1.01 (0.99, 1.03)
**Cheese intake**	<1/week	1 to 4/week	5/week to 1.4/day	≥1.5/day		
Direct update	1.00	0.97 (0.93, 1.01)	1.07 (1.03, 1.12)	1.19 (1.06, 1.33)	<0.001	1.10 (1.05, 1.14)
Stop updating CVD and cancer	1.00	0.96 (0.92, 1.00)	1.01 (0.98, 1.05)	1.08 (0.96, 1.20)	0.004	1.07 (1.03, 1.11)
Stop updating + HTHC	1.00	0.96 (0.92, 1.00)	1.00 (0.98, 1.03)	1.02 (0.92, 1.13)	0.41	1.03 (0.99, 1.07)
**Yogurt**	<1/month	1 to 3/month	1/week	≥2/week		
Direct update	1.00	1.01 (0.97, 1.06)	0.96 (0.92, 1.01)	0.91 (0.86, 0.96)	<0.001	0.83 (0.76, 0.92)
Stop updating CVD and cancer	1.00	1.00 (0.96, 1.05)	0.93 (0.89, 0.97)	0.88 (0.83, 0.93)	<0.001	0.83 (0.75, 0.92)
Stop updating + HTHC	1.00	0.97 (0.93, 1.02)	0.92 (0.88, 0.97)	0.87 (0.82, 0.91)	<0.001	0.86 (0.78, 0.94)
**Ice cream**	<1/month	1 to 3/month	1/week	≥2/week		
Direct update	1.00	0.85 (0.82, 0.89)	0.77 (0.73, 0.81)	0.68 (0.63, 0.72)	<0.001	0.73 (0.66, 0.81)
Stop updating CVD and cancer	1.00	0.87 (0.84, 0.91)	0.78 (0.74, 0.82)	0.74 (0.70, 0.79)	<0.001	0.78 (0.71, 0.86)
Stop updating + HTHC	1.00	0.96 (0.92, 1.00)	0.89 (0.85, 0.94)	0.89 (0.84, 0.94)	<0.001	0.89 (0.83, 0.96)
**Whole milk**	<1/month	1 to 3/month	1/week	≥2/week		
Direct update	1.00	1.03 (0.98, 1.10)	0.99 (0.94, 1.05)	1.12 (1.05, 1.19)	<0.001	1.10 (1.03, 1.17)
Stop updating CVD and cancer	1.00	1.01 (0.96, 1.07)	1.00 (0.94, 1.05)	1.08 (1.02, 1.14)	0.02	1.10 (1.04, 1.16)
Stop updating + HTHC	1.00	0.96 (0.90, 1.01)	1.04 (0.98, 1.11)	1.03 (0.98, 1.08)	0.15	1.03 (0.99, 1.07)

^a^ *P*-trend was calculated by assigning median values to each quintile and was treated as continuous variable; ^b^direct update: cumulative averages of dairy intakes were calculated without stopping updating diets. All multivariate models were adjusted for age (continuous), BMI (eight categories), total energy intake (quintiles), smoking, physical activity, alcohol consumption, menopausal status, race, diabetes family history, baseline hypertension, hypercholesterolemia, trans-fat intake, glycemic load, red and processed meat intake, nuts intake, SSB intake, and coffee intake, and other dairy types for individual dairy types; ^c^HTHC: hypertension and hypercholesterolemia. BMI, body mass index; CI, confidence interval; CVD, cardiovascular disease; HR, hazard ratio; SSB, sugar-sweetened beverages.

**Figure 2:**
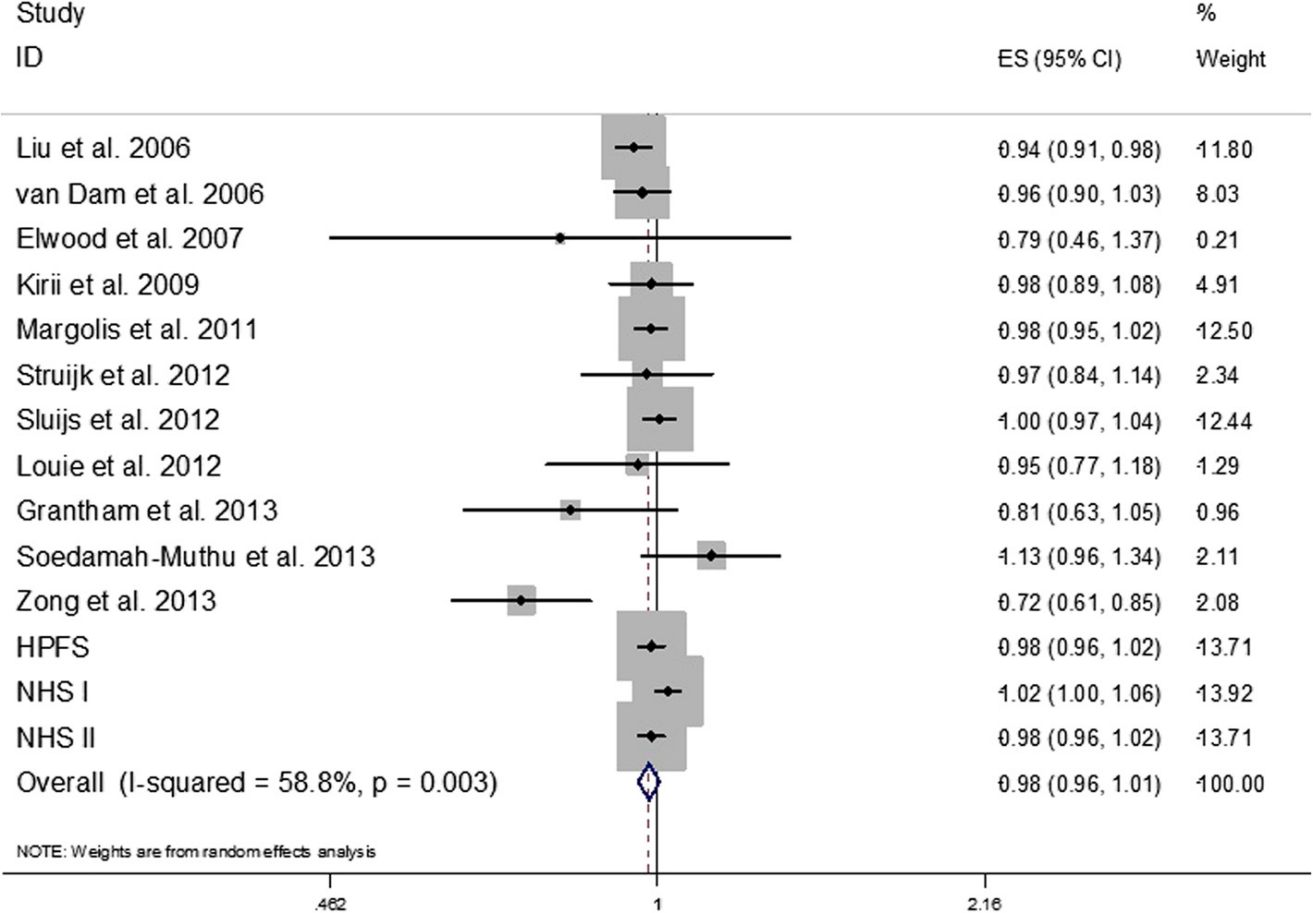
**HRs for a serving total dairy consumption per day and type 2 diabetes.** The RR of each study is represented by a square, and the size of the square represents the weight of each study of the overall estimate. The 95% CIs are represented by the horizontal lines, and the diamond represents the overall estimate and its 95% CI. HPFS, Health Professional Follow-Up Study; NHS, Nurses’ Health Study. CI, confidence interval; HRs, hazard ratios; RR; relative risk.

**Figure 3:**
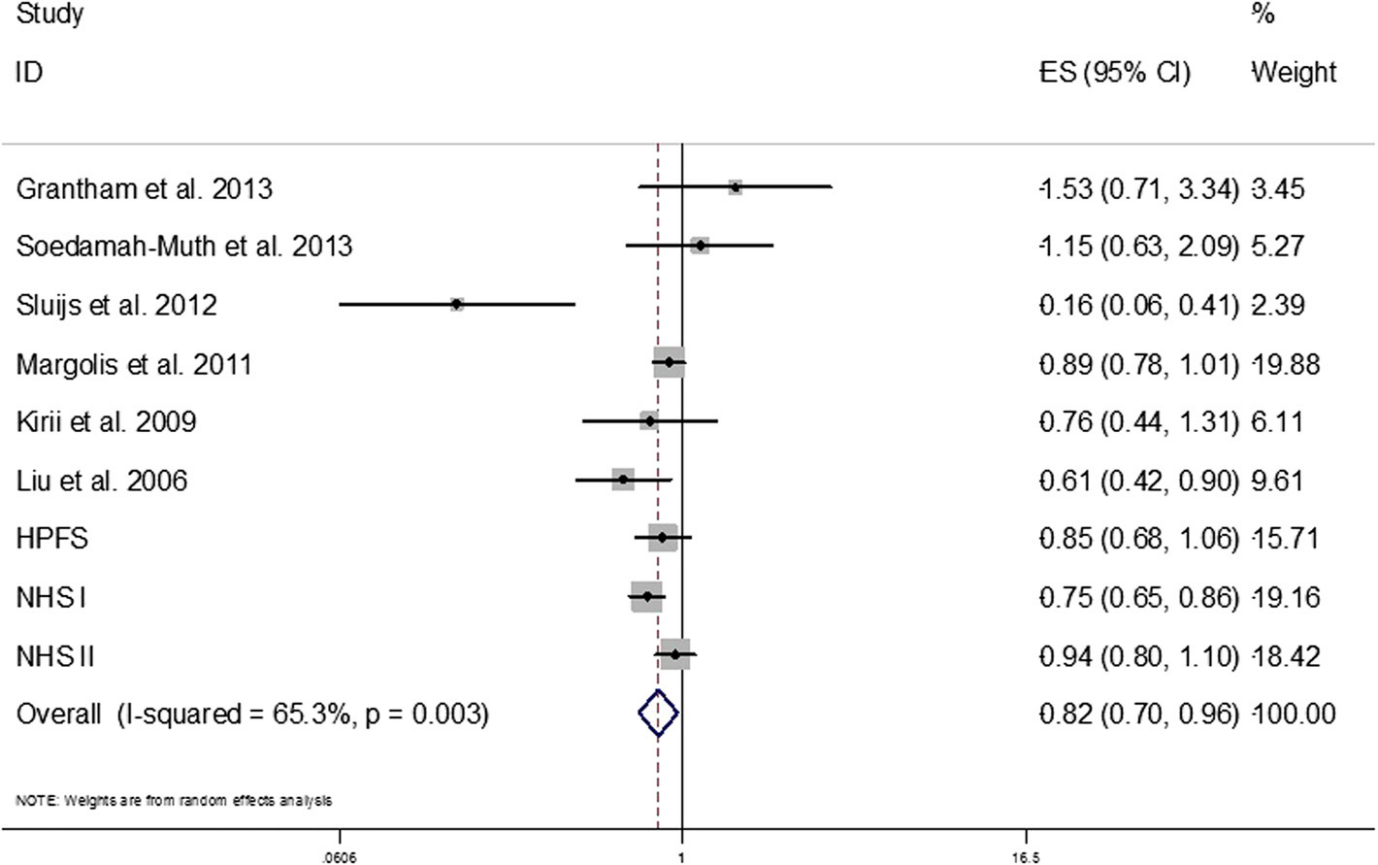
**HRs for a serving yogurt consumption per day and type 2 diabetes.** The RR of each study is represented by a square, and the size of the square represents the weight of each study of the overall estimate. The 95% CIs are represented by the horizontal lines, and the diamond represents the overall estimate and its 95% CI. HPFS, Health Professional Follow-Up Study; NHS, Nurses’ Health Study. CI, confidence interval; HRs, hazard ratios; RR, relative risk.

By incorporating our new results from the three cohorts together with the findings of previous studies, we conducted an updated meta-analysis. Our updated search on MEDLINE and EMBASE found 513 potential citations, of which one study [30] met the inclusion criteria, in addition to the citations in the two previous meta-analyses. Therefore, a total of eleven prospective studies [30-40] for total dairy and six [32-34,36-38] for yogurt were included in our updated meta-analysis, along with results from our current analysis. The characteristics of the included studies are shown in Additional file 1: Table S3. Total dairy intake was not significantly associated with risk of T2D whereas yogurt intake was associated with a significantly lower risk of T2D, as shown in Figures 2 and 3. Significant heterogeneity was shown for both total dairy (*I*^2^ = 58.8%; *P* = 0.003) and yogurt (*I*^2^ = 63.2%; *P* = 0.005). The RRs (95% CIs) from the random-effects model for one serving of total dairy intake and one serving/day yogurt intake were 0.98 (0.96, 1.01) and 0.82 (0.70, 0.96), respectively. The RRs (95% CIs) from the fixed-effects model for one serving/day of total dairy intake and one serving/day yogurt intake were 0.99 (0.98, 1.00) and 0.84 (0.78, 0.90), respectively.

After two studies [30,31] that did not adjust for total energy intake and other main dietary confounders were excluded, the heterogeneity for total dairy decreased (*I*^2^ = 35.6%; *P* = 0.11); additionally, heterogeneity for yogurt was reduced when one study [36] was removed (*I*^2^ = 40.7%; *P* = 0.063). Both exclusions did not change the summary RRs materially. On the basis of a funnel plot [see Additional file 1: Figure S2] and Begg’s test, no significant publication bias was shown for the association between total dairy (*P* = 0.19) or yogurt (*P* = 0.92) intake and risk of T2D.

We also classified studies according to duration of follow-up as short-term (≤10 years) or long-term (>10 years). Total dairy consumption was marginally significantly associated with a lower T2D risk in the short-term studies (RR: 0.95, 95%CI: 0.91, 1.00) but not in the long-term studies (RR: 1.00, 95%CI: 0.98, 1.02). Yogurt consumption was associated with a lower T2D risk in both short-term (RR: 0.86, 95%CI: 0.69, 1.08) and long-term studies (RR: 0.76, 95%CI: 0.58, 0.98).

## Discussion

In three prospective cohorts of US men and women, we found that intakes of total dairy products were not significantly associated with the risk of T2D, but higher consumption of yogurt was significantly associated with a lower risk. An updated meta-analysis of our cohorts and published literature suggest a marginally lower risk of T2D with higher dairy consumption and a consistent inverse association between yogurt consumption and T2D risk.

Dairy is a complex food with many bioactive compounds that have divergent health effects, and its association with T2D has attracted much attention [5]. Our results on total dairy intake and T2D risk are consistent with some [33,34,36,37,40], but not all previous studies. Total dairy consumption was associated with a lower risk of T2D in our earlier investigations in HPFS [11] and NHS [12], but not in NHS II [13]. The reason for the discrepancy between our earlier and current results is probably due to longer follow-up (10 more years) of the NHS and HPFS cohorts, and our meta-analysis suggests that potential benefits of dairy were less evident with long-term follow-up. For yogurt consumption, we observed a consistent and robust inverse association with T2D in our cohorts and the meta-analysis. A previous meta-analysis [8] reported a similar but nonsignificant risk estimate of development of T2D associated with 200 g yogurt consumption with an RR of 0.78 (95%CI: 0.60, 1.02). Our updated meta-analysis suggested that each one serving/day yogurt increase was significantly associated with a 18% lower risk. Surprisingly, consumption of ice cream was inversely associated with T2D risk whereas skim milk was associated with higher T2D risk; however, these associations either became null or attenuated in further analysis when we stopped updating dietary information after self-reported diagnosis of hypertension or hypercholesterolemia during the follow-up. Since consumption of ice cream was decreased but consumption of skim milk was increased after diagnosis of hypertension or hypercholesterolemia in our three cohorts [see Additional file 1: Table S4], reverse causation may explain the findings that did not take into account changes in diet after diagnosis of these conditions.

Certain components in dairy products, such as calcium, vitamin D, magnesium, lactose and dairy protein, have been suggested to have a favorable impact on metabolic factors, including body weight, hypertension [41,42] and glucose homeostasis [43]. Calcium supplement has been showed to have a small but significant reduction in body weight over a placebo in a recent meta-analysis [44] of seven trials, but the largest study [45] included in the meta-analysis did not find a significant effect of two-year calcium supplement use compared to the placebo. Conjugated linoleic acid, created by bacteria in the gut of ruminants, has been shown to reduce body weight in animals [46]. However, findings from randomized trials did not provide clear support for a role of dairy products in weight reduction [47]. Milk proteins, such as whey, may have insulinotropic properties with a relatively low glycemic load (GL), which may improve glucose tolerance [48]. Circulating trans-palmitoleate concentrations [7] have been inversely associated with insulin resistance, atherogenic dyslipidemia and incident diabetes. Whole-fat dairy product consumption was strongly associated with higher trans-palmitoleate which may offset the unfavorable effect of saturated fat in high-fat dairy product intake.

Several mechanisms may explain the inverse association between yogurt intake and risk of T2D. Probiotic bacteria have been shown to improve lipid profile and antioxidant status in T2D patients [49,50] and have beneficial effects on cholesterol levels [51]. In addition, our previous study [10] of the three cohorts showed that increased consumption of yogurt was inversely associated with weight gain. However, adjusting for BMI in the multivariate model did not alter the inverse association between yogurt intake and T2D risk.

The strengths of the current study include a large sample size, high rates of follow-up and repeated assessments of dietary and lifestyle variables. The current study was subject to several limitations as well. First, our study populations primarily consisted of health professionals of European ancestry. Although the homogeneity of socioeconomic status helps reduce confounding, the observed associations may not be generalizable to other populations. However, the relatively high educational status is an advantage because high quality and reliable data can be collected from our study participants. Second, because diet was assessed by FFQs, some measurement error of dairy intake assessment is inevitable. However, the FFQs used in these studies were validated against multiple diet records, and reasonable correlation coefficients between these assessments of dairy intake were observed. Moreover, we calculated cumulative averages for dietary variables to minimize the random measurement error caused by within-person variation and to accommodate diet changes over time. Nonetheless, since we did not specifically assess types or brands of yogurt consumed by the participants, it is difficult to attribute the observed benefits to various components of yogurt. Lastly, because of the observational nature of our cohorts, the observed associations do not necessarily mean causation; although we adjusted for established and potential risk factors for T2D, unmeasured and residual confounding is still possible. This is especially true for yogurt consumption, which is typically associated with a healthy diet and lifestyle.

## Conclusions

We found that higher intake of yogurt is associated with a reduced risk of T2D, whereas other dairy foods and consumption of total dairy are not appreciably associated with incidence of T2D. The consistent findings for yogurt suggest that it can be incorporated into a healthy dietary pattern. However, randomized clinical trials are warranted to further examine the causal effects of yogurt consumption as well as probiotics on body weight and insulin resistance.

## Additional file

Additional file 1:
**Supplemental Methods. Table S1:** Baseline age-adjusted characteristics of participants in the 3 cohorts according to categories of yogurt consumption^*a*^. **Table S2:** Type 2 diabetes according to total dairy intake: stratified analyses. **Table S3:** Type 2 diabetes according to yogurt consumption stratified by baseline BMI. **Table S4:** Changes in dairy products intake after hypertension and hypercholesterolemia in the 3 cohorts. **Table S5:** Multivariate relative Risk (RR) of type 2 diabetes among men and women according to different types of cheese. **Table S6:** Characteristics of studies included in the meta-analysis of the association of dairy intake with type 2 diabetes. **Figure S1:** Age-standardized trends of dairy foods consumption in three cohorts. **Figure S2A:** Test for publication bias for the association between total dairy intake and type 2 diabetes. **Figure S2B:** Test for publication bias for the association between yogurt intake and type 2 diabetes.

## Abbreviations

BMI: body mass index
CI: confidence interval
FFQ: food frequency questionnaire
HPFS: Health Professionals Follow-up Study
HR: hazard ratio
NHS: Nurses’ Health Study
RR: relative risk
SSBs: sugar sweetened beverages
T2D: type 2 diabetes
