# Supplementary material for: Dairy consumption and risk of type 2 diabetes: 3 cohorts of US adults and an updated meta-analysis
Source: BMC Med. 2014 Nov 25;12:215. doi: 10.1186/s12916-014-0215-1 (PMC4243376; doi:10.1186/s12916-014-0215-1)
Supplement: Additional file 1: — Supplemental Methods. Table S1: Baseline age-adjusted characteristics of participants in the 3 cohorts according to categories of yogurt consumptiona. Table S2: Type 2 diabetes according to total dairy intake: stratified analyses. Table S3: Type 2 diabetes according to yogurt consumption stratified by baseline BMI. Table S4: Changes in dairy products intake after hypertension and hypercholesterolemia in the 3 cohorts. Table S5: Multivariate relative Risk (RR) of type 2 diabetes among men and women according to different types of cheese. Table S6: Characteristics of studies included in the meta-analysis of the association of dairy intake with type 2 diabetes. Figure S1: Age-standardized trends of dairy foods consumption in three cohorts. Figure S2A: Test for publication bias for the association between total dairy intake and type 2 diabetes. Figure S2B: Test for publication bias for the association between yogurt intake and type 2 diabetes. [file 12916_2014_215_MOESM1_ESM.docx]

**Additional file**

**Supplemental Methods**

MEDLINE search query

#1

"dairy products"[Mesh] OR "dairy"[tiab] OR "milk"[tiab] OR "calcium"[tiab] OR "cheese"[tiab] OR "yogurt"[tiab]

#2

"Diabetes Mellitus"[Mesh] OR "Diabetes Mell itus, Type 2"[Mesh] OR "diabetes*"[tiab] OR "diabetic*"[tiab]

#3

(#1 AND #2)) AND ("2013/06/05"[Date - Publication] : "3000"[Date - Publication]

EMBASE search query

#1

'dairy products'/exp OR 'milk':ab OR 'calcium':ab OR 'cheese':ab OR 'yogurt':ab AND [embase]/lim AND [5-6-2013]/sd NOT [4-10-2013]/sd

#2

'diabetes mellitus'/exp OR 'diabetes':ab OR 'diabetic':ab AND [embase]/lim AND [5-6-2013]/sd NOT [4-10-2013]/sd

3

#1 AND #2 AND [embase]/lim

**Supplemental Table S1. Baseline age-adjusted characteristics of participants in the 3 cohorts according to categories of yogurt consumption*^a^***

|  | HPFS (1986) | | | | NHS I (1980) | | | | NHS II (1991) | | | |
| --- | --- | --- | --- | --- | --- | --- | --- | --- | --- | --- | --- | --- |
| Characteristics | <1s/mo (n=22394) | 1-3s/mo (n=8046) | 1s/wk (n=6481) | ≥2s/wk (n=4558) | <1s/mo (n=24764) | 1-3s/mo (n=13880) | 1s/wk (n=17665) | ≥2s/wk (n=11503) | <1s/mo (n=22049) | 1-3s/mo  (n=18098) | 1s/wk  (n=25712) | ≥2s/wk (n=20025) |
| Total dairy intake (servings/d) | 1.79(1.38)*^b^* | 1.90(1.29) | 2.11(1.34) | 2.45(1.50) | 1.75(0.98) | 1.93(0.93) | 2.18(0.91) | 2.58(0.98) | 1.88(1.40) | 2.13(1.40) | 2.34(1.41) | 2.71(1.50) |
| Age (y) | 54.4(9.6) | 51.4(9.0) | 50.6(8.8) | 52.1(9.5) | 46.2(7.2) | 46.1(7.2) | 46.1(7.2) | 46.1(7.2) | 36.1(4.7) | 35.9(4.7) | 36.0(4.7) | 36.3(4.6) |
| Physical activity (MET-h/wk) | 19.1(26.5) | 22.1(30.5) | 24.6(33.9) | 25.9(30.0) | 12.0(17.7) | 13.7(21.0) | 15.0(19.9) | 17.8(24.3) | 18.1(25.2) | 19.2(25.2) | 21.2(26.8) | 25.6(31.8) |
| BMI (kg/m^2^) | 25.0(4.9) | 25.0(4.8) | 24.7(5.0) | 24.6(4.9) | 24.3(4.5) | 24.4(4.4) | 24.2(4.2) | 23.9(4.1) | 24.7(5.6) | 24.6(5.2) | 24.4(5.0) | 24.2(4.9) |
| Race, white (%) | 94.9 | 95.0 | 95.5 | 95.5 | 97.9 | 97.6 | 98.0 | 98.1 | 95.3 | 96.0 | 97.1 | 97.6 |
| Current smoker (%) | 12.9 | 6.9 | 5.2 | 4.3 | 35.9 | 25.4 | 22.0 | 21.6 | 17.0 | 11.8 | 10.3 | 9.4 |
| Hypertension (%) | 20.2 | 19.2 | 17.3 | 17.3 | 16.1 | 15.5 | 13.5 | 13.2 | 6.5 | 6.2 | 5.6 | 5.3 |
| High cholesterol (%) | 9.9 | 11.0 | 10.6 | 10.8 | 5.2 | 5.3 | 5.0 | 5.3 | 15.3 | 14.5 | 13.3 | 13.5 |
| Family history of diabetes (%) | 23.1 | 24.7 | 25.9 | 25.1 | 28.9 | 29.1 | 28.7 | 27.4 | 33.4 | 33.2 | 34.3 | 33.6 |
| Postmenopausal (%) | NA | NA | NA | NA | 32.2 | 31.3 | 30.7 | 30.9 | 3.9 | 3.5 | 3.4 | 3.4 |
| Current menopausal hormone use (%)*^c^* | NA | NA | NA | NA | 20.7 | 21.8 | 21.6 | 22.3 | 71.8 | 74 | 72.6 | 73.5 |
| Current oral conceptive use (%) | NA | NA | NA | NA | NA | NA | NA | NA | 11.0 | 10.9 | 11.1 | 10.8 |
| Total energy (Kcal/d) | 1957(618) | 1975(611) | 2054(611) | 2148(643) | 1653(430) | 1675(410) | 1733(406) | 1831(418) | 1695(546) | 1746(541) | 1804(532) | 1906(546) |
| Alcohol (g/d) | 12.3(16.4) | 10.8(14.6) | 10.1(13.7) | 9.9(13.3) | 6.5(10.5) | 5.9(8.9) | 5.8(8.1) | 5.6(7.8) | 2.9(6.4) | 3.1(6.2) | 3.3(6.1) | 3.3(5.7) |
| Cereal fiber (g/d) | 5.5(3.7) | 6.1(4.0) | 6.3(4.1) | 6.7(4.3) | 4.4(2.1) | 4.9(2.0) | 5.2(2.0) | 5.2(2.0) | 5.1(2.9) | 5.6(2.9) | 5.8(3.0) | 6.0(3.4) |
| Glycemic load | 121.9(26.6) | 125.4(25.3) | 127.1(23.7) | 129.9(24.5) | 100.8(18.6) | 102.2(16.4) | 103.6(14.6) | 104.1(14.2) | 121.7(23.9) | 121.3(21.5) | 121.4(20.5) | 121.6(20.0) |
| Polyunsaturated to saturated fat ratio | 0.6(0.2) | 0.6(0.2) | 0.6(0.2) | 0.6(0.2) | 0.5(0.2) | 0.6(0.2) | 0.6(0.1) | 0.6(0.2) | 0.5(0.2) | 0.5(0.2) | 0.5(0.2) | 0.5(0.2) |
| Trans fat (% of total energy) | 3.0(1.2) | 2.8(1.1) | 2.6(1.0) | 2.4(1.0) | 1.9(0.5) | 1.8(0.5) | 1.6(0.4) | 1.5(0.4) | 1.8(0.7) | 1.7(0.6) | 1.6(0.6) | 1.5(0.5) |
| Fruit and vegetables (servings/d) | 4.9(2.5) | 5.6(2.7) | 6.0(2.9) | 6.7(3.2) | 4.5(1.9) | 5.1(1.9) | 5.6(1.9) | 6.1(2.1) | 4.2(2.6) | 4.8(2.6) | 5.3(2.8) | 6.1(3.2) |
| red processed meat intake (servings/d) | 1.3(0.9) | 1.1(0.8) | 1.0(0.8) | 0.9(0.7) | 1.2(0.6) | 1.0(0.5) | 0.9(0.5) | 0.9(0.5) | 1.2(0.7) | 1.2(0.7) | 1.1(0.7) | 1.1(0.7) |
| Nuts intake (servings/d) | 0.44(0.62) | 0.47(0.62) | 0.51(0.61) | 0.55(0.67) | 0.13(0.18) | 0.15(0.18) | 0.17(0.20) | 0.19(0.22) | 0.07(0.17) | 0.08(0.19) | 0.09(0.19) | 0.10(0.25) |
| SSB intake (servings/d) | 0.42(0.69) | 0.33(0.56) | 0.30(0.49) | 0.29(0.52) | 0.37(0.57) | 0.28(0.42) | 0.24(0.36) | 0.22(0.34) | 0.64(1.04) | 0.48(0.84) | 0.42(0.75) | 0.35(0.68) |
| Coffee intake (servings/d) | 2.00(1.83) | 1.85(1.74) | 1.87(1.76) | 1.79(1.74) | 2.17(1.59) | 2.10(1.45) | 2.09(1.36) | 2.17(1.41) | 1.36(1.70) | 1.49(1.66) | 1.58(1.66) | 1.74(1.68) |

*^a^*Data were age standardized except for age. HPFS, Health Professionals Follow-Up Study; METs, metabolic equivalent; NA, not available; NHS, Nurses’ Health Study.

*^b^*Mean SD (all such values)

*^c^*Current menopausal hormone users among postmenopausal women.

**Supplemental Table S2. Type 2 diabetes according to total dairy intake: stratified analyses**

|  | Frequency of dairy consumption | | | | | *P*-trend*^b^* | HR (95% CI) for one serving/d |
| --- | --- | --- | --- | --- | --- | --- | --- |
|  | Q1*^a^* | Q2*^a^* | Q3*^a^* | Q4*^a^* | Q5*^a^* |  |  |
| **HPFS** |  |  |  |  |  |  |  |
| Stratified by age |  |  |  |  |  |  |  |
| <60y (1044 cases) | 1.00 | 1.07 (0.89, 1.29)*^3^* | 0.86 (0.70, 1.05) | 0.83 (0.68, 1.02) | 0.86 (0.69, 1.06) | 0.05 | 0.95 (0.89, 1.00) |
| ≥60y (2325 cases) | 1.00 | 1.09 (0.96, 1.25) | 1.10 (0.96, 1.25) | 1.08 (0.94, 1.23) | 1.05 (0.91, 1.21) | 0.84 | 1.00 (0.96, 1.03) |
| Stratified by BMI |  |  |  |  |  |  |  |
| <25 (956 cases) | 1.00 | 1.03 (0.84, 1.26) | 0.96 (0.78, 1.18) | 0.91 (0.74, 1.13) | 0.97 (0.79, 1.21) | 0.62 | 0.97 (0.92, 1.03) |
| ≥25 (2413 cases) | 1.00 | 1.11 (0.98, 1.27) | 1.03 (0.90, 1.18) | 1.04 (0.91, 1.19) | 0.99 (0.86, 1.14) | 0.49 | 0.99 (0.95, 1.02) |
| Stratified by vitamin D level |  |  |  |  |  |  |  |
| < 378 (1741 cases) | 1.00 | 1.08 (0.94, 1.24) | 1.06 (0.92, 1.22) | 0.93 (0.80, 1.09) | 0.93 (0.78, 1.11) | 0.15 | 0.95 (0.91, 1.00) |
| ≥ 378 (1628 cases) | 1.00 | 1.10 (0.92, 1.31) | 0.96 (0.80, 1.15) | 1.07 (0.90, 1.27) | 1.04 (0.87, 1.23) | 0.83 | 1.00 (0.96, 1.04) |
| Stratified by physical activity level |  |  |  |  |  |  |  |
| < 23 MET-h/wk (2117 cases) | 1.00 | 1.14 (1.00, 1.31) | 1.09 (0.94, 1.25) | 1.07 (0.93, 1.23) | 1.05 (0.90, 1.22) | 0.96 | 0.99 (0.96, 1.03) |
| ≥ 23 MET-h/wk (1252 cases) | 1.00 | 1.00 (0.83, 1.20) | 0.91 (0.75, 1.09) | 0.88 (0.73, 1.06) | 0.87 (0.71, 1.05) | 0.09 | 0.96 (0.91, 1.00) |
| Stratified by DM family history |  |  |  |  |  |  |  |
| Yes (1555 cases) | 1.00 | 1.01 (0.87, 1.17) | 0.94 (0.80, 1.09) | 0.99 (0.85, 1.15) | 0.93 (0.79, 1.09) | 0.34 | 0.98 (0.94, 1.02) |
| No (1814 cases) | 1.00 | 1.20 (1.02, 1.41) | 1.12 (0.95, 1.32) | 1.02 (0.85, 1.21) | 1.06 (0.89, 1.26) | 0.67 | 0.99 (0.94, 1.03) |
| **NHS** |  |  |  |  |  |  |  |
| Stratified by age |  |  |  |  |  |  |  |
| <60y (2796 cases) | 1.00 | 1.00 (0.89, 1.13) | 0.95 (0.83, 1.07) | 0.97 (0.85, 1.11) | 1.09 (0.95, 1.24) | 0.25 | 1.04 (0.99, 1.08) |
| ≥60y (5057 cases) | 1.00 | 0.99 (0.90, 1.08) | 0.99 (0.90, 1.08) | 1.06 (0.96, 1.17) | 1.01 (0.91, 1.12) | 0.56 | 1.00 (0.97, 1.04) |
| Stratified by BMI |  |  |  |  |  |  |  |
| <25 kg/m2 (1087 cases) | 1.00 | 1.20 (0.99, 1.44) | 1.06 (0.86, 1.30) | 1.24 (1.01, 1.52) | 1.14 (0.91, 1.41) | 0.32 | 1.04 (0.97, 1.12) |
| ≥25 kg/m2 (6766 cases) | 1.00 | 0.96 (0.89, 1.04) | 0.96 (0.89, 1.04) | 1.00 (0.92, 1.08) | 1.02 (0.93, 1.11) | 0.40 | 1.01 (0.98, 1.04) |
| Stratified by vitamin D level |  |  |  |  |  |  |  |
| <400 (4264 cases) | 1.00 | 0.98 (0.90, 1.07) | 0.95 (0.87, 1.05) | 1.05 (0.95, 1.16) | 1.12 (1.00, 1.27) | 0.05 | 1.05 (1.01, 1.09) |
| ≥400 (3589 cases) | 1.00 | 1.04 (0.91, 1.18) | 1.02 (0.90, 1.16) | 1.04 (0.92, 1.19) | 1.04 (0.91, 1.19) | 0.65 | 1.00 (0.96, 1.04) |
| Stratified by physical activity level |  |  |  |  |  |  |  |
| < 10 MET-h/wk (4943 cases) | 1.00 | 0.99 (0.90, 1.08) | 0.93 (0.85, 1.02) | 1.03 (0.93, 1.13) | 0.95 (0.86, 1.06) | 0.58 | 0.99 (0.96, 1.03) |
| ≥10 MET-h/wk (2910 cases) | 1.00 | 1.02 (0.90, 1.15) | 1.06 (0.93, 1.20) | 1.03 (0.91, 1.18) | 1.18 (1.03, 1.36) | 0.01 | 1.05 (1.00, 1.10) |
| Stratified by DM family history |  |  |  |  |  |  |  |
| Yes (4049 cases) | 1.00 | 0.96 (0.87, 1.06) | 0.96 (0.86, 1.06) | 0.98 (0.88, 1.09) | 1.00 (0.90, 1.12) | 0.78 | 1.00 (0.97, 1.04) |
| No (3804 cases) | 1.00 | 1.02 (0.92, 1.13) | 1.00 (0.89, 1.11) | 1.08 (0.96, 1.20) | 1.07 (0.95, 1.20) | 0.19 | 1.03 (0.99, 1.07) |
| **NHS II** |  |  |  |  |  |  |  |
| Stratified by age |  |  |  |  |  |  |  |
| <60y (3928 cases) | 1.00 | 1.04 (0.95, 1.15) | 1.04 (0.94, 1.15) | 1.00 (0.90, 1.11) | 0.98 (0.88, 1.10) | 0.48 | 0.98 (0.95, 1.01) |
| ≥60y (42 cases) | 1.00 | 0.82 (0.34, 1.94) | 0.63 (0.21, 1.88) | 0.21 (0.04, 1.01) | 0.58 (0.18, 1.81) | 0.15 | 0.96 (0.67, 1.37) |
| Stratified by BMI |  |  |  |  |  |  |  |
| <25 kg/m2 (235 cases) | 1.00 | 0.81 (0.56, 1.17) | 0.66 (0.44, 0.98) | 0.51 (0.32, 0.81) | 0.69 (0.44, 1.07) | 0.03 | 0.85 (0.74, 0.97) |
| ≥25 kg/m2 (3735 cases) | 1.00 | 1.09 (0.98, 1.20) | 1.08 (0.97, 1.20) | 1.04 (0.93, 1.15) | 1.02 (0.90, 1.14) | 0.77 | 0.99 (0.96, 1.02) |
| Stratified by vitamin D level |  |  |  |  |  |  |  |
| <355 (1874 cases) | 1.00 | 1.14 (1.01, 1.30) | 1.08 (0.94, 1.23) | 0.99 (0.85, 1.15) | 1.12 (0.94, 1.32) | 0.70 | 1.01 (0.96, 1.05) |
| ≥355 (1965 cases) | 1.00 | 0.95 (0.82, 1.10) | 0.98 (0.85, 1.14) | 0.97 (0.83, 1.12) | 0.91 (0.77, 1.06) | 0.25 | 0.97 (0.93, 1.01) |
| Stratified by physical activity level |  |  |  |  |  |  |  |
| <12 MET-h/wk (2331 cases) | 1.00 | 1.11 (0.98, 1.25) | 1.04 (0.91, 1.18) | 1.00 (0.87, 1.15) | 0.98 (0.85, 1.13) | 0.36 | 0.96 (0.92, 1.00) |
| ≥12 MET-h/wk (1639 cases) | 1.00 | 0.97 (0.83, 1.13) | 1.02 (0.87, 1.20) | 0.96 (0.82, 1.13) | 0.98 (0.82, 1.17) | 0.81 | 1.01 (0.97, 1.06) |
| Stratified by DM family history |  |  |  |  |  |  |  |
| Yes (1496 cases) | 1.00 | 1.13 (0.96, 1.33) | 1.18 (1.00, 1.39) | 1.14 (0.96, 1.36) | 1.13 (0.94, 1.36) | 0.34 | 1.02 (0.97, 1.07) |
| No (2474 cases) | 1.00 | 1.02 (0.91, 1.15) | 0.97 (0.86, 1.10) | 0.92 (0.80, 1.05) | 0.91 (0.79, 1.05) | 0.07 | 0.95 (0.92, 0.99) |

*^a^*Q is quintile

*^b^P*-trend was calculated by assigning median values to each quintile and was treated as continuous variable.

*^c^*All multivariate models were adjusted for age (continuous), BMI (8 categories), total energy intake (quintiles), race, smoking, physical activity, alcohol consumption, menopausal status and menopausal hormone use (NHS I and II participants only), oral contraceptive use (NHS II participants only), diabetes family history, hypertension, hypercholesterolemia, trans-fat intake, glycemic load, red processed meat intake, nuts intake, SSB intake, and coffee intake, and other dairy types for individual dairy types.

**Supplemental Table S3. Type 2 diabetes according to yogurt consumption stratified by baseline BMI**

|  | Yogurt Intake (servings) | | | | *P* for trend*^a^* | HR (95% CI) for one serving/d |
| --- | --- | --- | --- | --- | --- | --- |
| Variable | Category 1 | Category 2 | Category 3 | Category 4 |  |  |
| **BMI ≤25** | <1/mo | 1-3/mo | 1/wk | ≥2/wk |  |  |
| HPFS |  |  |  |  |  |  |
| Multivariate Model*^b^* | 1.00 | 0.76 (0.61, 0.94) | 0.83 (0.66, 1.03) | 0.89 (0.69, 1.15) | 0.39 | 1.00 (0.66, 1.52) |
| NHS |  |  |  |  |  |  |
| Multivariate Model | 1.00 | 0.91 (0.82, 1.01) | 0.85 (0.76, 0.94) | 0.75 (0.66, 0.85) | <0.001 | 0.66 (0.52, 0.84) |
| NHS II |  |  |  |  |  |  |
| Multivariate Model | 1.00 | 0.91 (0.74, 1.14) | 0.97 (0.79, 1.18) | 0.82 (0.65, 1.04) | 0.12 | 0.77 (0.52, 1.14) |
| Pooled |  |  |  |  |  |  |
| Multivariate Model | 1.00 | 0.88 (0.81, 0.96) | 0.86 (0.79, 0.94) | 0.79 (0.71, 0.87) | <0.001 | 0.74 (0.62, 0.90) |
| **BMI >25** | <1/mo | 1-3/mo | 1/wk | ≥2/wk |  |  |
| HPFS |  |  |  |  |  |  |
| Multivariate Model | 1.00 | 1.04 (0.94, 1.15) | 0.91 (0.81, 1.03) | 0.98 (0.84, 1.13) | 0.55 | 0.81 (0.62, 1.05) |
| NHS |  |  |  |  |  |  |
| Multivariate Model | 1.00 | 1.07 (1.00, 1.16) | 0.94 (0.87, 1.01) | 0.88 (0.80, 0.97) | <0.001 | 0.79 (0.67, 0.95) |
| NHS II |  |  |  |  |  |  |
| Multivariate Model | 1.00 | 1.02 (0.92, 1.12) | 1.01 (0.92, 1.11) | 0.93 (0.83, 1.03) | 0.11 | 0.99 (0.83, 1.18) |
| Pooled |  |  |  |  |  |  |
| Multivariate Model | 1.00 | 1.05 (1.00, 1.10) | 0.96 (0.91, 1.01) | 0.92 (0.86, 0.98) | <0.001 | 0.87 (0.78, 0.97) |

*^a^P*-trend was calculated by assigning median values to each quintile and was treated as continuous variable.

*^b^*All multivariate models were adjusted for age (continuous), BMI (8 categories), total energy intake (quintiles), race, smoking, physical activity, alcohol consumption, menopausal status and menopausal hormone use (NHS I and II participants only), oral contraceptive use (NHS II participants only), diabetes family history, hypertension, hypercholesterolemia, trans-fat intake, glycemic load, red processed meat intake, nuts intake, SSB intake, and coffee intake, and other dairy types for individual dairy types.

**Supplemental Table S4. Changes in dairy products intake after hypertension and hypercholesterolemia in the 3 cohorts**

|  | Yogurt | | Skim milk | | Cheese | | Ice-cream | | Whole milk | |
| --- | --- | --- | --- | --- | --- | --- | --- | --- | --- | --- |
|  | Change (serving/d) | *P-*value | Change (serving/d) | *P-*value | Change (serving/d) | *P-*value | Change (serving/d) | *P-*value | Change (serving/d) | *P-*value |
| HPFS |  |  |  |  |  |  |  |  |  |  |
| HT*^a^* | -0.003(-0.009, 0.003)^2^ | 0.27 | 0.007 (-0.019, 0.032) | 0.62 | 0.004 (-0.008, 0.017) | 0.50 | -0.002 (-0.008, 0.003) | 0.41 | -0.004 (-0.011, 0.003) | 0.29 |
| HC*^a^* | -0.002 (-0.006, 0.002) | 0.38 | 0.023 (0.006, 0.041) | 0.009 | -0.062(-0.070, -0.053) | <0.001 | -0.029 (-0.033, -0.025) | <0.001 | -0.027 (-0.032, -0.022) | <0.001 |
| NHS |  |  |  |  |  |  |  |  |  |  |
| HT | -0.009 (-0.015, -0.004) | <0.001 | -0.002 (-0.018, 0.014) | 0.79 | -0.001(-0.010, 0.008) | 0.83 | -0.007(-0.011, -0.004) | 0.001 | -0.003(-0.008, 0.001) | 0.17 |
| HC | -0.006 (-0.009, -0.002) | 0.002 | 0.035 (0.023, 0.046) | <0.001 | -0.062(-0.069, -0.056) | <0.001 | -0.022(-0.025, -0.019) | <0.001 | -0.020(-0.023, -0.017) | <0.001 |
| NHS II |  |  |  |  |  |  |  |  |  |  |
| HT | -0.007(-0.012, -0.001) | 0.03 | -0.036(-0.050, -0.021) | <0.001 | 0.008 (-0.002, 0.018) | 0.11 | -0.004(-0.006, -0.001) | 0.01 | 0.001(-0.002, 0.004) | 0.49 |
| HC | -0.002(-0.007, 0.002) | 0.34 | -0.014(-0.026, -0.002) | 0.023 | -0.032(-0.039, -0.024) | <0.001 | -0.006(-0.008, -0.004) | <0.001 | -0.004(-0.007, -0.002) | <0.001 |

*^a^*HT: hypertension; HC: hypercholesterolemia

*^b^*Results were generated from generalized linear models on changes in dairy products intake from one FFQ circle to next FFQ circle. Covariates included cancer diagnosis, coronary heart disease diagnosis, stroke diagnosis, hypertension diagnosis, hypercholesterolemia diagnosis, calorie intake, FFQ circles, dairy products intake of last FFQ circle.

**Supplemental Table S5. Multivariate relative Risk (RR) of type 2 diabetes among men and women according to different types of cheese**

|  | Dairy Intake (servings) | | | | *P* for trend*^a^* | HR (95% CI) for one serving/d |
| --- | --- | --- | --- | --- | --- | --- |
| Variable | Category 1 | Category 2 | Category 3 | Category 4 |  |  |
| **Cottage cheese** | <1/mo | 1-3/mo | 1/wk | ≥2/wk |  |  |
| HPFS |  |  |  |  |  |  |
| Cases/person-years | 1144/288506 | 1068/269986 | 776/165732 | 376/74221 |  |  |
| Multivariate Model*^b^* | 1.00 | 0.95 (0.87, 1.04) | 1.04 (0.94, 1.15) | 1.07 (0.95, 1.22) | 0.15 | 0.91 (0.74, 1.12) |
| NHS |  |  |  |  |  |  |
| Cases/person-years | 1539/325407 | 2347/508729 | 2396/475845 | 1022/191062 |  |  |
| Multivariate Model | 1.00 | 0.98 (0.92, 1.05) | 1.03 (0.97, 1.11) | 1.04 (0.95, 1.13) | 0.17 | 1.09 (0.94, 1.25) |
| NHS II |  |  |  |  |  |  |
| Cases/person-years | 1534/579794 | 1372/523609 | 827/260855 | 218/66983 |  |  |
| Multivariate Model | 1.00 | 1 (0.92, 1.07) | 1.12 (1.02, 1.23) | 1.01 (0.87, 1.17) | 0.27 | 1.01 (0.78, 1.32) |
| Pool |  |  |  |  |  |  |
| Multivariate Model | 1.00 | 0.98 (0.94, 1.02) | 1.06 (1.01, 1.11) | 1.04 (0.98, 1.11) | 0.03 | 1.02 (0.92, 1.14) |
| **Cream cheese** | <1/mo | 1-3/mo | 1/wk | ≥2/wk |  |  |
| HPFS |  |  |  |  |  |  |
| Cases/person-years | 1753/443462 | 1028/242837 | 399/82421 | 184/29725 |  |  |
| Multivariate Model | 1.00 | 1.06 (0.98, 1.15) | 1.17 (1.04, 1.31) | 1.34 (1.14, 1.57) | <0.001 | 1.34 (1.03, 1.74) |
| NHS |  |  |  |  |  |  |
| Cases/person-years | 3636/765816 | 2315/479419 | 1105/215101 | 248/40708 |  |  |
| Multivariate Model | 1.00 | 1.09 (1.03, 1.15) | 1.16 (1.08, 1.25) | 1.25 (1.1, 1.43) | <0.001 | 1.59 (1.27, 1.97) |
| NHS II |  |  |  |  |  |  |
| Cases/person-years | 1707/641608 | 1473/539713 | 610/208114 | 161/41807 |  |  |
| Multivariate Model | 1.00 | 1.05 (0.98, 1.13) | 1.09 (0.99, 1.20) | 1.28 (1.09, 1.51) | 0.002 | 1.34 (1.03, 1.74) |
| Pool |  |  |  |  |  |  |
| Multivariate Model | 1.00 | 1.07 (1.03, 1.11) | 1.14 (1.08, 1.20) | 1.29 (1.18, 1.40) | <0.001 | 1.44 (1.25, 1.66) |
| **Other cheese** | <1/mo | 1-3/mo | 1/wk | ≥2/wk |  |  |
| HPFS |  |  |  |  |  |  |
| Cases/person-years | 147/37750 | 358/99477 | 967/243278 | 1892/417940 |  |  |
| Multivariate Model | 1.00 | 0.92 (0.76, 1.12) | 1.04 (0.86, 1.24) | 1.06 (0.88, 1.27) | 0.11 | 1.04 (0.95, 1.13) |
| NHS |  |  |  |  |  |  |
| Cases/person-years | 483/73055 | 570/123043 | 2304/482574 | 3947/822372 |  |  |
| Multivariate Model | 1.00 | 0.93 (0.82, 1.06) | 0.95 (0.85, 1.06) | 0.91 (0.81, 1.01) | 0.05 | 1.00 (0.92, 1.08) |
| NHS II |  |  |  |  |  |  |
| Cases/person-years | 74/25149 | 417/136722 | 1038/396284 | 2422/873086 |  |  |
| Multivariate Model | 1.00 | 0.97 (0.76, 1.24) | 0.85 (0.67, 1.08) | 0.90 (0.71, 1.14) | 0.90 | 0.99 (0.91, 1.09) |
| Pool |  |  |  |  |  |  |
| Multivariate Model | 1.00 | 0.93 (0.85, 1.03) | 0.96 (0.88, 1.04) | 0.94 (0.86, 1.02) | 0.44 | 1.01 (0.96, 1.06) |

*^1^*

*^a^P*-trend was calculated by assigning median values to each quintile and was treated as continuous variable.

*^b^*All multivariate models were adjusted for age (continuous), BMI (8 categories), total energy intake (quintiles), race, smoking, physical activity, alcohol consumption, menopausal status and menopausal hormone use (NHS I and II participants only), oral contraceptive use (NHS II participants only), diabetes family history, hypertension, hypercholesterolemia, trans-fat intake, glycemic load, red processed meat intake, nuts intake, SSB intake, and coffee intake, and other dairy types for individual dairy types.

**Supplemental Table S6:** Characteristics of studies included in the meta-analysis of the association of dairy intake with type 2 diabetes

| **References** | **Cohort name** | **Total No. of cases and participants** | **Follow-up years** | **Age at baseline** | **Sex** | **Diet assessment** | **Diabetes assessment** | **Adjusted covariates** |
| --- | --- | --- | --- | --- | --- | --- | --- | --- |
| Liu et al. 2006 | Women's Health Study, USA | 1603 cases, 37,183 participants | 1993-2003, 10 years | 55 | Female | Validated FFQ*^a^*, 131 food and beverage items | Diagnostic criteria of ADA*^a^*, based on self-reporting, 3 complementary approaches to validate the cases | Age, total energy intake, randomized treatment assignment, FH DM*^a^*, smoking status, BMI, hypercholesterolemia, hypertension, physical activity, hormones, alcohol, dietary fiber, total fat, GL*^a^* |
| van Dam et al. 2006 | Black Women's Health Study, USA | 1964 cases, 41186 participants | 1995-2003, 8 years | 21-69 | Female | Validated FFQ, 68 food items | Self-report | Age, total energy intake, alcohol, BMI, smoking status, strenuous physicial activity, parental history of DM*^a^*, education, coffee, sugar-sweetened soft drinks, processed meat, red meat, whole grains |
| Elwood et al. 2007 | Caerphilly Prospective Study, UK | 41 cases, 640 participants | 1979/1983-NA, 20 years | 45-59 | Male | 7-d weighted records | Self-report | Age, smoking, BMI, social class |
| Kirii et al. 2009 | Japan Public Health Center-based Prospective Study, Japan | 1114 cases, 59796 participants | 1995/1998-2000/2003, 5 years | 40-69 | Both | Validated FFQ, 147 food and beverage items | Self-report | Age, area, BMI, FH DM, smoking status, alcohol intake, history of hypertension, exercise frequency, coffee consumption, energy-adjusted Mg, total energy intake |
| Margolis et al. 2011 | Women's Health Initiative, USA | 3946 cases, 82,076 participants | 1994/1998-2005, 7.9 years | 50-79 | Female | FFQ, >300 foods and beverages | Self-report, confirmed by review of medical records | Age, race-ethnicity, total energy intake, income, education, BMI, smoking, alcohol intake, FH DM, HRT*^a^*, SBP*^a^*, DBP*^a^*, physical activity, interaction of low-fat dairy×BMI, interaction of yogurt×time |
| Louie et al. 2012 | The Blue Mountains Eye Study, Australia | 145 cases, 1824 participants | 1992/1994-2002/2004, 10 years | 63.5 | Both | Validated FFQ, 145 food items | Self-report, taking medication for T2D, fasting blood glucose>7.0mmol/L | Age, sex, smoking status, physical activity, GL, vegetable fiber, total energy, FH DM, SBP*^a^*, BMI, HDL, total cholesterol, TG*^a^* |
| Sluijs et al. 2012 | EPIC-Interact Study, Europe*^a^* | 10,694 cases, 24,475 participants | 1991-2007, 11.7 years | 52 | Both | Validated FFQ, 24-h recall | Self-report, primary care registers, secondary care registers, medication use(drug registers), hospital admissions, and mortality data | Age, center, sex, BMI, education, smoking status, physical activity, intake of alcohol, fruit and vegetables, red meat, processed meat, sugar-sweetened soft drinks, coffee, cereals, cereal products, energy |
| Struijk et al. 2012 | The Inter99 Study, Denmark | 214 cases, 5953 participants | 1999/2001-2006, 5 years | 30-60 | Both | Validated FFQ, 198 food items | 75 g OGTT*^a^* | Age, sex, intervention group, FH DM, education, physical activity, smoking status, intakes of alcohol, whole-grain cereal, meat, fish, coffee, tea, fruit, vegetables, energy, change in diet from baseline to 5-y follow-up, waist circumference |
| Grantham et al. 2013 | The Australian Diabetes Obesity and Lifestyle Study, Australia | 209 cases, 5582 participants | 1999/2000-2004/2005, 5 years | 52 | Both | FFQ, 121 food items | 75 g OGTT | Age, sex, energy intake, FH DM, education, physical activity, smoking status, TG, HDL cholesterol, SBP, waist circumference, hip circumference |
| Soedamah-Muthu et al. 2013 | The Whitehall II Prospective Study, UK | 273 cases, 4526 participants | 1985/1988-2009, 10 years | 56 | Both | FFQ, 114 food items | Self-report, and 75 g OGTT | Age, ethnicity, employment grade, smoking, BMI, physical activity, FH CHD*^a^*/hypertension, intakes of alcohol, fruit and vegetables, bread, meat, fish, coffee, tea, total energy |
| Zong et al. 2013 | the Nutrition and Health of Aging Population in China | 504 cases, 2,091 participants | 2005-2011, 6 years | 50-70 | Both | 74-item FFQ | Self-report, taking medication for T2D*^a^*, fasting blood glucose>7.0mmol/L | age, sex, region, smoking, family history of diabetes, BMI, and dietary fiber intake |
| Chen et al. 2014 | Health Professional Follow-up Study, USA | 3364 cases, 41,436 participants | 1986-2010, 24 years | 40-75 | Male | Updated validated FFQ | Self-report + Appendix questionnaires | age, BMI, total energy intake, smoking, physical activity, alcohol consumption, menopausal status, race, diabetes family history, hypertension, hypercholesterolemia, trans-fat intake, glycemic load, red processed meat intake, nuts intake, SSB intake, and coffee intake |
| Chen et al. 2014 | Nurses' Health Study I, USA | 7841 cases, 67,138 participants | 1980-2010, 30 years | 34-59 | Female | Updated validated FFQ | Self-report + Appendix questionnaires | Same as above plus postmenopausal status and menopausal hormone use |
| Chen et al. 2014 | Nurses' Health Study II, USA | 3951 cases, 85,884 participants | 1991-2009, 18 years | 27-44 | Female | Updated validated FFQ | Self-report + Appendix questionnaires | Same as above plus postmenopausal status and menopausal hormone use, and oral contraceptive use |

*^a^* ADA, ; CHD, coronary heart disease; DBP, ; DM, diabetes mellitus; T2D, type 2 diabetes; EPIC, European Investigation into Cancer and Nutrition; FFQ, food frequency questionnaire; FH, family history; GL, glycemic load; HRT, ; OGTT; TG, triacylglycerol; SBP, systolic blood pressure;

**Supplement figure 1. Age-standardized trends of dairy foods consumption in three cohorts**

**Supplemental Figure S2A**: Test for publication bias for the association between total dairy intake and type 2 diabetes

Tests for publication bias: Begg’s test, *P*=0.19; Egger’s test, *P*=0.57

**Supplemental Figure S2B**: Test for publication bias for the association between yogurt intake and type 2 diabetes

Tests for publication bias: Begg’s test, *P*=0.92; Egger’s test, *P*=0.44
